# Distinct Roles of Directional and Positional Experience in De Novo Visuomotor Learning

**DOI:** 10.1523/ENEURO.0001-26.2026

**Published:** 2026-09-01

**Authors:** Tomoya Kawano, Shota Hagio

**Affiliations:** 1 Laboratory of Motor Control and Learning, Graduate School of Human and Environmental Studies, Kyoto University, Kyoto 606-8501, Japan; 2 Research Fellow of Japan Society for the Promotion of Science, Tokyo 102-0083, Japan

**Keywords:** motor learning

## Abstract

Humans can flexibly acquire entirely new sensorimotor mappings, a process known as de novo motor learning. A central challenge in de novo motor learning is that the learner must discover a viable solution from scratch within a highly redundant control space, without predefined task constraints. Understanding what types of sensorimotor information contribute to the formation of accurate motor behavior in such situations is therefore critical for explaining how novel sensorimotor skills are acquired. While previous studies have suggested that novel visuomotor mappings can be formed based on movement direction and target position, it remains unclear how these two types of information contribute to the learning process. To address this question, we trained 25 human participants (13 males and 12 females) to learn arbitrary joystick-to-cursor mapping. We then employed a generalization paradigm to selectively restrict learning experience to either movement direction or target position. Three distinct target conditions were designed: one emphasized target position (P), another emphasized movement direction (D), and a third (P&D) encouraged learning of both components separately. Results showed direction experience reduced initial direction error, whereas the lack of position experience was associated with increased final position error. However, in the P&D condition, combining these experiences did not yield additive generalization. Instead, endpoint accuracy was positively correlated with the alignment between direction- and position-based outputs in the redundant control space. These results suggest that accurate formation of a novel sensorimotor map depends on the coordinated use of directional and positional experiences.

## Significance Statement

How do humans build entirely new sensorimotor relationships from scratch? This study examined how distinct sensorimotor experiences (movement direction and target position) contribute to the acquisition of a novel joystick-to-cursor mapping. By isolating these experiences, we found that direction experience improved movement initiation, while the lack of position experience reduced movement termination accuracy. However, combining these experiences did not lead to more accurate movements as a whole. Instead, the accuracy was related to how well directional and positional joystick outputs were aligned in redundant control space. These findings suggest that de novo motor learning requires the coordinated use of directional and positional information.

## Introduction

Humans possess a remarkable ability to learn arbitrary sensorimotor maps, a process known as de novo motor learning (Krakauer et al., 2019). This capability underlies many everyday skills, such as playing musical instruments or using unfamiliar tools. For example, when first operating a remote-controlled device (e.g., a drone or robotic manipulator), individuals must learn how their motor commands applied to a handheld device translate into movements of a spatially separate external effector. De novo motor learning refers to the process of establishing this novel relationship (i.e., forming a new sensorimotor map). Previous studies have primarily focused on motor adaptation, where pre-existing sensorimotor maps are recalibrated in response to environmental changes (Shadmehr and Mussa-Ivaldi, 1994; Krakauer et al., 2000). However, information regarding how such sensorimotor maps are built from scratch remains limited.

To experimentally induce de novo motor learning, researchers have devised a variety of tasks. A commonly used approach is to create an arbitrary mapping between the position of a body part and on-screen cursor position (Mosier et al., 2005; Haith et al., 2022; Kawano et al., 2024). For example, in a bimanual task, vertical movements of one hand control horizontal cursor movements and vice versa, forcing participants to acquire a novel visuomotor map through practice (Yang et al., 2021; Haith et al., 2022). While participants initially struggle to direct the cursor toward intended targets, they gradually acquire precise control with training. Although these tasks evoke de novo motor learning, how the brain constructs a new sensorimotor map remains poorly understood. Unlike motor adaptation, de novo motor learning requires establishing entirely new mappings between actions and sensory consequences. This distinction raises a fundamental question: what aspects of the sensorimotor experience does the brain rely on when constructing a novel sensorimotor mapping from scratch?

It has been proposed that the brain can represent visuomotor map based on two distinct types of information (Wang and Sainburg, 2005; Wu and Smith, 2013; Van der Graaff et al., 2017). One is to transform a movement direction into a change in hand posture (vector coding); and the other is to transform a target position into a specific hand posture (position coding). Several studies suggested that de novo motor learning relies on vector coding (Mosier et al., 2005; Ranganathan et al., 2013). Supporting this idea, it has been observed that the cursor trajectory tended to became straight even without explicit instruction (Mosier et al., 2005). In addition, the final hand posture at the target has been shown to depend on the movement direction toward the target (Ranganathan et al., 2013). Conversely, other findings suggested the involvement of position coding. Several studies have demonstrated that de novo motor learning tasks can be learned without continuous visual feedback of a cursor (Liu and Scheidt, 2008; Kim et al., 2024; Park et al., 2024). This finding implies that the brain can construct visuomotor maps using only information about the target position (i.e., position coding). Together, these findings suggest that the brain can build a novel visuomotor map using information about both movement direction and target position.

In this study, we aimed to elucidate how two types of information—movement direction and target position—contribute to de novo motor learning. To this end, we employed a generalization paradigm (Malfait et al., 2002; Donchin et al., 2003; Shadmehr, 2004), which allows us to dissociate the contributions of direction and position experiences by examining how restricted learning experience generalizes to untrained movements. We conducted two experiments. In Experiment 1, three distinct target conditions were designed to elicit generalization from experience with specific components: one target emphasized target position alone, another emphasized movement direction alone, and a third encouraged learning of both elements separately. We define “separately” as experiencing both the matched position and direction of an untrained movement through distinct training contexts, whereas “alone” refers to experiencing only one of these matched components. This experimental design is crucial for examining whether the sensorimotor system can integrate separate learning experiences. We reasoned that if direction and position information additively contribute to the construction of a sensorimotor map, then acquiring both components should lead to greater generalization than experiencing either component alone. Experiment 2 served as a control experiment in which a separate group of participants was trained only on one target condition. This experiment tested whether the generalization pattern observed in Experiment 1 was influenced by interleaving different target conditions within the same participants.

## Materials and Methods

### Experiment 1

#### Participants

Twenty-five right-handed individuals without a history of neurological or motor disorders were included in this study [13 males and 12 females; aged 20.8 ± 1.87 years, mean ± standard deviation (SD)]. All participants provided written informed consent after receiving a detailed description of the purpose, potential benefits, and risks of the experiment. One female participant was excluded due to failure to complete the whole task within the 1.5-h experiment time (complete up to the 12th block out of 20). All procedures used in this study were performed in accordance with the Declaration of Helsinki and were approved by the Ethics Committee for Human Experimentation at the Graduate School of Human and Environmental Studies, Kyoto University (24-H-15).

#### Experimental setup

The participants sat in front of two three-axis joysticks (Model: 9SA50RR-22-47; APEM) equipped on an adjustable table with a 27-in LCD monitor (1,920 × 1,080-pixel resolution, 165 Hz refresh rate; ASUS; Fig. 1*A*). Both joysticks were positioned symmetrically with respect to the participant’s midline, placed 30 cm forward from the face, and separated laterally by 22.5 cm. Participants could not see their hands, and their head motion was limited by a chinrest. Participants manipulated the two joysticks with both hands using three fingers (thumb, index, and middle finger). Each joystick measured 3-axis positions (i.e., *X*: horizontal, *Y*: vertical, and *Z*: roll axis), acquiring a total of 6 input signals in 16-bit at a sampling rate of 50 Hz. Both joysticks were spring-loaded, meaning they passively returned to the center position when the fingers were removed. We performed preprocessing on these inputs in real time using a custom LabVIEW program as follows: Initially, joystick inputs were normalized such that each axis could vary independently within the range of [ − 1, 1], where ±1 corresponded to ±14.4° tilt for the *X*- and *Y*-axes, and ±27° rotation for the *Z*-axis. Thereafter, the five-point moving average was applied to reduce the intrinsic noise of the joysticks. The post-processing 6-dimensional vector ***h***, representing the joystick positions, was linearly mapped onto the lateral and vertical cursor position ***p*** using the equation below (Mosier et al., 2005; Ranganathan et al., 2013; Choi et al., 2020; Fig. 1*B*):

$$\left[\begin{array}{c} x\\ y \end{array}\right]=\left[\begin{array}{cccc} a_{1,1} & a_{1,2} & \ldots & a_{1,6}\\ a_{2,1} & a_{2,2} & \ldots & a_{2,6} \end{array}\right]\times {\left[\begin{array}{ccccc} h_{1} & h_{2} & \ldots & h_{5} & h_{6} \end{array}\right]}^{T},$$

i.e., ***p*** = ***Ah***, where the mapping matrix ***A*** was determined from the calibration task (described below).

**Figure 1.**
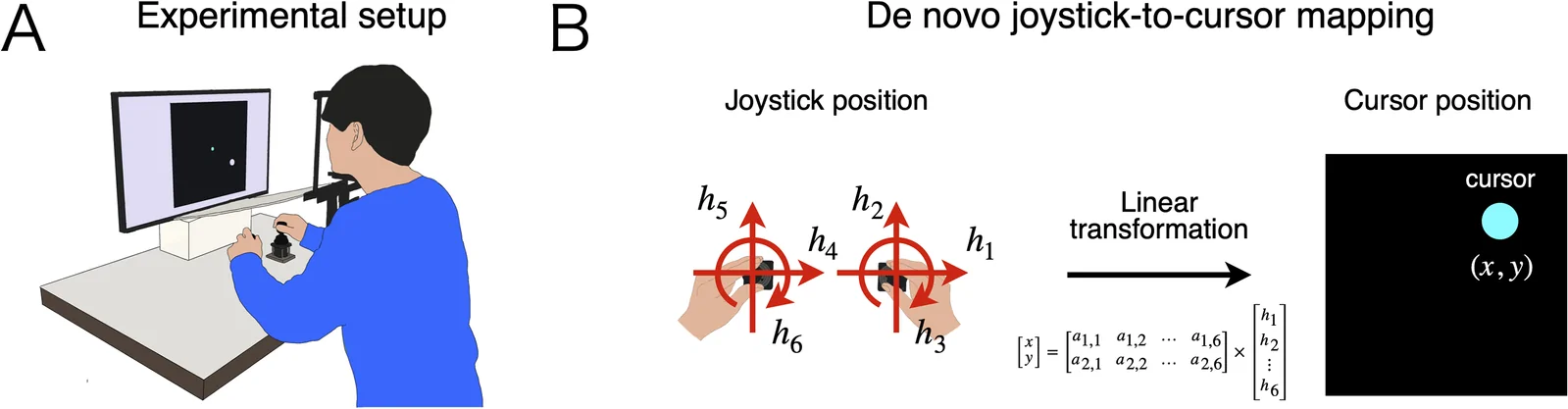
Experimental setup. ***A***, Participants manipulated two joysticks to control the on-screen cursor. ***B***, Cursor position was determined by the joystick position. Two joysticks measured a total of six input signals. The six-dimensional joystick position vector was linearly translated into the cursor position. Figure 1-1 shows the individual joystick-to-cursor mapping matrices (PC1 and PC2 loadings) for all participants.

10.1523/ENEURO.0001-26.2026.f1-1 Figure 1-1 Individual mapping matrices of all participants are shown. Blue and red bars represent PC1 and PC2, respectively. Download Figure 1-1, TIF file.

#### Experimental task

There were two tasks in the experiment: a calibration task and point-to-point reaching task.

##### Calibration task

To determine the mapping matrix ***A*** for each participant, calibration task was conducted. In this task, participants were instructed to move the joysticks freely for 1 min. To encourage participants to explore various joystick movements, we presented six bar graphs visualizing the real-time variance of joystick positions for each axis (Choi et al., 2020). The acquired calibration data was standardized for each axis to ensure that all axes contributed to the mapping matrix ***A***. Principal component analysis (PCA) was then performed on the standardized calibration data. PCA identifies orthogonal directions in the data that successively capture the largest variance, such that each principal component represents a dominant pattern of coordinated joystick movements. The first two principal components (PC1 and PC2) were used to form the first and second rows of the mapping matrix ***A***. Therefore, the scores of the PC1 and PC2 were converted to the lateral and vertical cursor position, respectively. We used first two PCs to avoid requiring participants to perform unfamiliar joystick movements in the following reaching task. Individual mapping matrices for all participants are shown in Figure 1-1.

##### Point-to-point reaching task

Participants were required to move the on-screen cursor (5 mm radius) to a series of targets (10 mm radius). During the task, targets were presented sequentially, with a sequence of trials referring to a group of consecutive reaches toward these targets (Fig. 2*A*). Details regarding the locations and order of the targets are described below (Experimental conditions). All trials were classified into either a training trial or a test trial. Each trial was defined as one reaching movement toward a single target. In the training trial, participants were given continuous visual feedback of the cursor. Based on this visual information, participants moved the cursor toward the target. When the cursor stayed within a target for 500 ms, it moved to a new location. At the end of every 2 or 3 training trials (referred to as a sequence of trials), the visual feedback of the cursor was removed and participants were instructed to relax the joysticks (i.e., release their hands so that the spring force passively returned each joystick to the center position) to reset the cursor position at the center of the workspace (Fig. 2*B*). The number of trials in each sequence differed across experimental conditions (see Experimental conditions). To control the amount of time spent across participants, a time limit of 12 s was imposed only on the final trial of each sequence. If the elapsed time exceeded 12 s, the trial was terminated and participants were instructed to relax the joysticks. Participants were instructed to move the cursor to the target as fast as possible. To motivate them to follow this instruction, we provided a score calculated based on the elapsed time after the start of the trial, which reflects how quickly they reached the target. The score was calculated as follows:

$$\text{score}=\left\{ \begin{array}{cc} 100 & \left(\text{time}\leq 1.5\right)\\ 100-\frac{100}{12-1.5}\times (\text{time}-1.5) & \left(1.5<\text{time}<12\right)\\ 0 & \left(\text{time}\geq 12\right) \end{array}\right..$$

In the test trial, the visual feedback of the cursor was removed to evaluate the open-loop movement learned during the training trial. The test trials were always initiated from the center of the workspace and directed toward one of the eight targets (Fig. 2*C*). The procedure for the test trial was as follows (Fig. 2*D*):

1. Participants positioned the cursor in the center of the workspace.
2. A target appeared, and 200 ms later the visual feedback of the cursor was removed. The 200 ms is the minimum reaction time for the visuomotor system (Georgopoulos et al., 1981; Soechting and Lacquaniti, 1983; Van Sonderen et al., 1988). This duration was set to allow participants to recognize the spatial relationship between the cursor and the target while preventing them from seeing the cursor movement afterwards.
3. Once the participants started moving the joysticks (onset) and then stopped them (offset), the trial ended. The onset was identified when the joystick tangential velocity (i.e., the time derivative of the 6D joystick position, computed as the Euclidean norm of the difference vector divided by the time interval) exceeded a threshold of 0.5 for 10 consecutive time steps. The offset was defined as the time when the joystick tangential velocity fell below a threshold of 0.5 after the onset. After the offset, participants were instructed to relax the joysticks.
4. The visual feedback of the cursor was restored after the joysticks returned to the center position.

To encourage participants to perform open-loop movement, temporal criteria on their behavior was imposed. If the time between the start of the trial and the offset exceeded 2 s, participants were provided with a warning message, “stop a little earlier.” To prevent participants from misinterpreting this message as spatial feedback (e.g., stopping short of the target), we explicitly instructed them beforehand that this warning indicated the movement duration was too long, and we encouraged them to complete their movement in a single, continuous motion. The test targets were located in eight directions at intervals of $45^{\circ }$ and a distance of 8 cm (Fig. 2*C*). Among the eight targets, four targets ( $0^{\circ }$, $90^{\circ }$, $180^{\circ }$, and $270^{\circ }$) were assigned to four different conditions. The assignment of conditions to these four targets was counterbalanced across participants. These targets were then used to implement the different experimental conditions described below.

**Figure 2.**
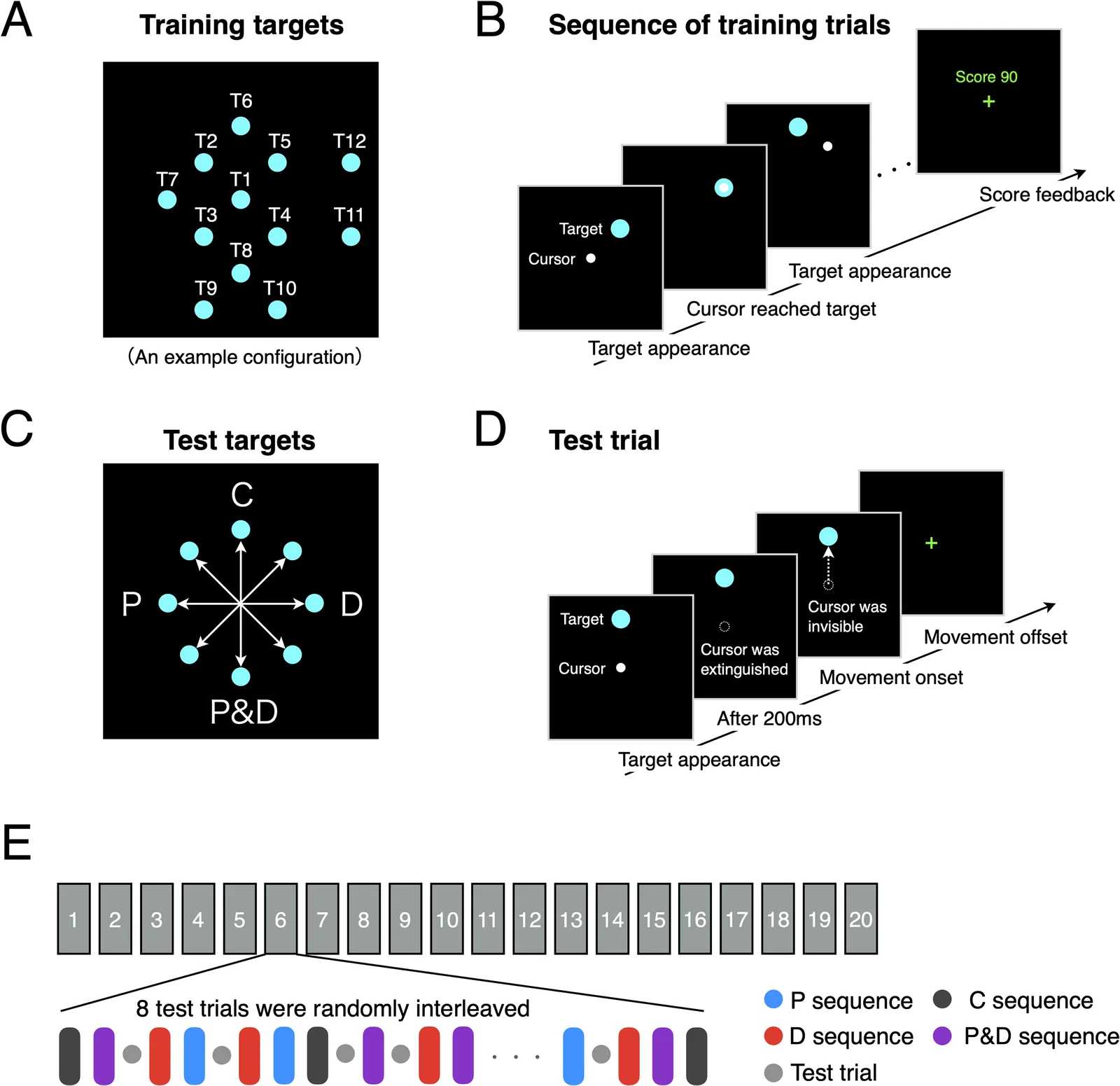
Experimental design. ***A***, The target presented in training trials consist of 12 targets, labeled T1–T12. This configuration illustrates one example of the possible arrangements. ***B***, The participants performed sequence of training trials to learn visuomotor maps. ***C***, The test targets were located in eight directions at intervals of $45^{\circ }$, and the start position was the center of the workspace. The assignment of conditions to the four test targets (C, P, D, and P&D) was counterbalanced across participants; the configuration shown here is one example. ***D***, The participants performed test trials to help measure the amount of visuomotor map learning. ***E***, The task consists of 20 blocks. In each block there are 20 sequences of training trials (rounded rectangle) and 8 test trials (gray circle). The sequences for each condition were presented in a pseudorandom order every four sequences. In block 1, test trials were presented at the beginning of the block. In the subsequent blocks, test trials were randomly interleaved between sequences. The four colors in the sequences (rounded rectangle) represent the differences in experimental conditions (black: Control, blue: Position, red: Direction, and purple: Position and Direction).

#### Experimental conditions

We designed four experimental conditions to test how learning generalized across untrained movements. To clarify how movement direction and target position contribute to the acquisition of a de novo visuomotor map, we employed a generalization paradigm (Malfait et al., 2002; Donchin et al., 2003; Shadmehr, 2004). This approach makes it possible to disentangle the respective roles of direction and position by testing how learning under restricted experience transfers to novel, untrained movements. Within this framework, we defined four conditions that varied in the type of information provided. The Control (C) condition involved direct practice of the test movement, serving as a baseline. The Position (P) condition exposed participants only to the final position of the test movement, while the Direction (D) condition exposed them only to movement direction. The Position and Direction (P&D) condition combined these two components, allowing participants to experience target position and movement direction separately. The aim of this experimental design is to evaluate how well learning generalizes to the P, D, and P&D conditions relative to the common baseline provided by the C condition, in which the test movement is directly trained. Figure 2*C* shows an example configuration of the test targets for the four conditions. The following explanations will be based on this configuration. There are 12 potential targets that could be presented in training trials (Fig. 2*A*, T1–T12). Participants were trained to move between these targets.

##### Control condition

In this condition, the movement in the test trial was a movement upward from the center of the workspace (Fig. 2*C*). This condition served as a baseline to assess the amount of learning when participants were directly trained on the test movement. To this end, participants were trained to perform the same upward movement as in the test trial. The training was conducted using a sequence of three trials. Initially, the cursor was located at T1. Then, targets were presented in the sequence of either T2→T1→T6 or T5→T1→T6 (Fig. 2*A*). The inclusion of the T2→T1 (or T5→T1) movements was intended to balance the number of movements toward the targets (T2–T5) surrounding the test target. These consecutive three trials are referred to as the C (Control) sequence.

##### Position condition

In this condition, the movement in the test trial was a movement to the left from the center of the workspace (Fig. 2*C*). During training, the target position was kept consistent while the movement direction was varied. To achieve this, they were trained to move to the same position, but from a different direction. The training was conducted using a sequence of two trials. Initially, the cursor was located at T1. Then, targets were presented in the sequence of either T2→T7 or T3→T7 (Fig. 2*A*). Importantly, T7 was the same as the test target of this condition (Fig. 2*C*). These consecutive two trials are referred to as the P (Position) sequence.

##### Direction condition

In this condition, the movement in the test trial was a movement to the right from the center of the workspace (Fig. 2*C*). During training, the movement direction was kept consistent while the target position was varied. To this end, they were trained to move from the same direction to different positions. The training was conducted using a sequence of two trials. Initially, the cursor was located at T1. Then, targets were presented in the sequence of either T5→T12 or T4→T11 (Fig. 2*A*). Crucially, T5→T12 and T4→T11 movements matched the movement direction of the test trial (Fig. 2*C*). This consecutive two trials are referred to as the D (Direction) sequence.

##### Position and direction condition

In this condition, the movement in the test trial was a movement downward from the center of the workspace (Fig. 2*C*). Participants were allowed to separately experience both the position and direction of the movement. To this end, participants were trained using both the P- and D-sequences. Initially, the cursor was located at T1. Then, targets were presented in the sequence of either T3→T8, T3→T9, T4→T8, or T4→T10 (Fig. 2*A*). Essentially, T8 was the same as the test target of this condition. Additionally, T3→T9 and T4→T10 movements matched the movement direction of the test trial (Fig. 2*C*). These consecutive two trials are referred to as the P&D (Position and Direction) sequence. If the effects of direction and position experience additively contribute to the acquisition of a de novo sensorimotor map, learning both components separately would yield greater generalization than learning either component alone (P condition and D condition).

#### Experimental procedure

The experimental task consisted of 20 blocks (Fig. 2*E*). Each block consisted of 20 training sequences and 8 test trials (Fig. 2*E*). The sequences for each condition were presented in a pseudorandom order every four sequences. In block 1, test trials were presented at the beginning of the block to test movements with minimal training effects. In the subsequent blocks, test trials were randomly interleaved between sequences. The rest period between blocks was 30 s, with a longer 120 s rest period provided every five blocks.

#### Data analysis

All data were analyzed using MATLAB R2025a (MathWorks, Inc.). We recorded cursor position, joystick position, and joystick tangential velocity at 50 Hz. The joystick tangential velocity was low-pass filtered (5 Hz) using a fourth-order Butterworth filter.

##### Task performance

The task performance in the training trials was assessed by movement time. The movement time was the time elapsed from movement onset to the end of the trial. The movement onset was defined as the time when joystick tangential velocity exceeded a threshold of 0.5 for 10 consecutive time steps. In the test trial, we characterized each reaching movement by separating it into two continuous phases: movement initiation and movement termination. To quantify the accuracy of these phases, we computed the initial direction error and the final position error, respectively. The final position error was calculated as the Euclidean distance between the final cursor position and the target position. The initial direction error was quantified as the absolute angle between the direction from the center of the workspace to the cursor position at the time when the joystick tangential velocity reached its first peak after movement onset and the direction from the center of the workspace to the target position. The peak of the joystick tangential velocity was identified using the MATLAB function findpeaks, with the argument MinPeakProminence set to 5% of the maximum velocity in each trial.

##### Position- and direction-based visuomotor outputs in joystick control space

In the P&D condition, the effects of position and direction experiences appear additive in the workspace since the direction experience matches the movement direction of the test trial and the position experience matches the target position (Fig. 5*A*). However, in the joystick control space, which includes task-irrelevant dimensions, visuomotor outputs based on direction and position are not necessarily aligned (Fig. 5*B*). We therefore tested how the degree of alignment between position- and direction-based visuomotor outputs influenced generalization in the P&D condition. To this end, we calculated how target positions or movement directions were transformed into joystick positions or joystick movement directions, respectively. The former was defined as the position-based visuomotor output (**VO**_pos_), and the latter as the direction-based visuomotor output (**VO**_dir_). To estimate **VO**_pos_, we averaged the final joystick positions across trials directed to the same target position. Similarly, to estimate **VO**_dir_, we calculated joystick movement vectors as the difference between the final and initial joystick positions in each trial, and then averaged these vectors across trials with the same movement direction in the workspace. For this analysis, we used training trials from the last five blocks. Because the number of trials for averaging was small (12 or 13), a bootstrap procedure with 1,000 resamples was used to obtain stable estimates of these averages. To quantify the alignment between the two outputs, we calculated the angle between **VO**_dir_ and **VO**_pos_ as $\arccos\;\left(\frac{{VO}_{\mathrm{dir}}\cdot {VO}_{\mathrm{pos}}}{\left\|{VO}_{\mathrm{dir}}\|\|{VO}_{\mathrm{pos}}\right\|}\right)$, where a smaller value indicates greater alignment. Finally, to examine whether this alignment explains individual differences in the final position error in the P&D condition while controlling for individual differences in movement initiation, we performed a linear regression at the participant level with the alignment and initial direction error as predictors of the final position error.

#### Outlier criteria

To avoid outlier influenced bias during hypothesis testing, outliers were removed using the quartile method. Specifically, this procedure was applied to the group data pooled across all participants within each condition. First, the first quartile (Q1) and the third quartile (Q3), which represent the 25th and 75th percentiles of the data, respectively, were calculated. Thereafter the interquartile range (IQR) was calculated by subtracting Q1 from Q3:

IQR=Q3−Q1.

Next, the lower- and upper bound for outliers were calculated:

$$\begin{array}{rl} & \text{Lower bound}=Q1-1.5\times \text{IQR},\\ & \text{Upper bound}=Q3+1.5\times \text{IQR}. \end{array}$$

Any data points outside this range were considered outliers. Outliers were identified separately for the initial direction error and the final position error, as these measures quantify distinct phases of the movement (initiation and termination). Accordingly, a trial that is extreme in one measure is not necessarily anomalous in the other.

#### Statistics

All statistical analyses were calculated using R version 4.2.2 (Rstudio, Inc.). Data from 24 participants were analyzed. To investigate the effects of condition and trial on the error in last 5 test trials (either final position error or initial direction error), we conducted a linear mixed-effects model analysis using the lme4 package in R (Bates et al., 2015). For the initial direction error, the fixed effects included condition and trial. For the final position error, we additionally included initial direction error as a covariate. This was motivated by the fact that the final cursor position is geometrically influenced by the initial movement direction; statistically controlling for initial direction error therefore allowed us to isolate the contribution of each condition to movement termination beyond what was already explained by movement initiation. First, outliers within each condition were excluded using the quartile method (a total of 3.1% and 2.1% of the data was removed for initial direction error and final position error, respectively). After outlier removal, the analyses included 465 observations for initial direction error and 470 observations for final position error. The random effects included both random intercepts and random slopes for participants. Statistical significance of the fixed effects was determined using Type III *F*-tests. Post hoc pairwise comparisons were conducted using *t*-tests on estimated marginal means derived from the linear mixed-effects models (using the emmeans package in R), and *p*-values were adjusted for multiple comparisons using the Benjamini–Hochberg procedure. In all statistical analyses, a *p*-value of less than 0.05 was considered statistically significant. Cohen’s *d* was computed as effect size. An overview of the data structure and statistical tests used in this study is provided in Table 1.

**Table 1.** Statistical table summarizing data structure and statistical tests

	Data structure	Type of test	95% CI
a	Normal distribution	Type III ANOVA	–
b	Normal distribution	Type III ANOVA	–
c	Normal distribution	Post hoc pairwise *t*-test	[−16.9, −1.46]
d	Normal distribution	Post hoc pairwise *t*-test	[−9.33, 5.04]
e	Normal distribution	Post hoc pairwise *t*-test	[−13.0, 2.91]
f	Normal distribution	Post hoc pairwise *t*-test	[−10.5, 4.77]
g	Normal distribution	Post hoc pairwise *t*-test	[−1.52, 15.6]
h	Normal distribution	Type III ANOVA	–
i	Normal distribution	Type III ANOVA	–
j	Normal distribution	Type III ANOVA	–
k	Normal distribution	Post hoc pairwise *t*-test	[−1.86, −0.064]
l	Normal distribution	Post hoc pairwise *t*-test	[−1.89, −0.347]
m	Normal distribution	Post hoc pairwise *t*-test	[−1.01, 0.696]
n	Normal distribution	Linear regression coefficient	[0.0153, 0.0849]
o	Normal distribution	*t*-test	[−16.0, −5.16]
p	Normal distribution	*t*-test	[−4.71, 0.77]
q	Normal distribution	*t*-test	[−13.6, 0.77]
r	Normal distribution	*t*-test	[−2.70, −1.37]
s	Normal distribution	*t*-test	[−3.02, −1.27]
t	Normal distribution	*t*-test	[−1.4, 0.532]

Lowercase letters (a–t) correspond to the superscript letters reported in the Results that identify each statistical analysis.

### Experiment 2

To assess whether interleaving the four training conditions within each block in the Experiment 1 introduced interference among them, we conducted Experiment 2. In Experiment 2, a separate group of participants was trained exclusively on the D (Direction) sequence.

#### Participants

Twenty-four right-handed individuals without a history of neurological or motor disorders participated in this control experiment (10 males and 14 females; aged 20 ± 2.82 years, mean ± SD). The sample size was matched to that of the Experiment 1. All participants provided written informed consent.

#### Task

The experimental setup, and calibration procedure were identical to those of the Experiment 1. The only differences were the composition of training targets and training sequences within each block (Fig. 6*A*). Participants were trained exclusively on the D (Direction) sequence. Accordingly, the training targets were reduced to five (Fig. 6*A*, T1–T5). The two trained movements (T4 → T5 and T2 → T3) were directionally matched to the test direction, in the same manner as the D condition of the Experiment 1. The assignment of the D condition to one of the four cardinal test directions was counterbalanced across participants. The task consisted of 10 blocks (Fig. 6*B*). Each block consisted of 20 D training sequences and 8 test trials that were randomly interleaved between the sequences. Of the 8 test trials per block, 4 were directed to the trained target positions (2 trials for each of the two trained movements, T2 → T3 and T4 → T5), which is defined as C condition. The remaining 4 trials were directed to the D condition test target (Fig. 6*A* right).

#### Data analysis

The initial direction error and final position error were quantified using the same methods as in Experiment 1. Because participants in Experiment 2 were trained exclusively on the D-sequence with 20 D sequences per block (compared with 5 per block in Experiment 1), the same amount of practice accumulated within fewer blocks.

To align practice experience across conditions and experiments, two matched reference points were defined in Experiment 2. The mid-training reference for the D condition (D_mid_) was established at the end of block 5 (Fig. 6*B*), at which point participants had completed 100 D sequences, matching the total D-sequence experience of the D condition in Experiment 1. The last-training reference for C condition (C_last_) was measured at the end of block 10 (Fig. 6*B*). Because the two trained movements (T2 → T3 and T4 → T5) were each presented 10 times per block, participants had accumulated 100 direct practices for each by block 10, matching the direct practice amount of the C condition in Experiment 1 (100 C sequences). A comparison between D_mid_ and C_last_ therefore assessed whether directional training alone produced performance comparable to that achieved through an equivalent amount of direct practice. In addition, D_mid_ was compared with the D condition of Experiment 1 to verify whether matching the total amount of D-sequence practice across different experimental designs yielded comparable task performance.

To examine the effect of additional directional training, a late-training reference for the D condition (D_last_) was defined at the end of block 10 (Fig. 6*B*), by which point participants had completed 200 D sequences. Comparing D_last_ with C_last_ allowed us to determine whether further practice enables generalization.

#### Statistics

All statistical analyses were performed using R, following the same framework as in Experiment 1. For each reference point (C_last_, D_mid_, and D_last_), five test trials at the corresponding stage of training were used. As in Experiment 1, outliers within each condition were excluded using the quartile (IQR) method. The proportion of data removed for the initial direction error was 3.3%, 1.7%, and 3.3% in C_last_, D_mid_, and D_last_, respectively, and 11.7%, 1.7%, and 2.5% for the final position error. Both errors were analyzed using linear mixed-effects models, and pairwise comparisons were conducted on the estimated marginal means using the emmeans package. Cohen’s *d* was computed as the effect size. The initial direction error was included as a covariate when analyzing the final position error, as in Experiment 1. In the within-subject comparisons within Experiment 2 (C_last_ vs D_mid_ and C_last_ vs D_last_), the models included participant-specific random intercepts and random slopes, whereas in the between-subject comparison between the D condition of Experiment 1 and D_mid_ in Experiment 2, only random intercepts were included.

## Results

### Experiment 1

#### Task performance improved in the training trials

Participants learned the de novo visuomotor task through repeated training. Figure 3*A* shows the cursor trajectories of a representative participant in training trials. In the early stages, the cursor movements were erratic under all conditions. In contrast, in the later stages, the cursor moved directly toward the target more efficiently. The movement time decreased as the training progressed, indicating that participants learned to move the cursor to the target more quickly (Fig. 3*B*).

**Figure 3.**
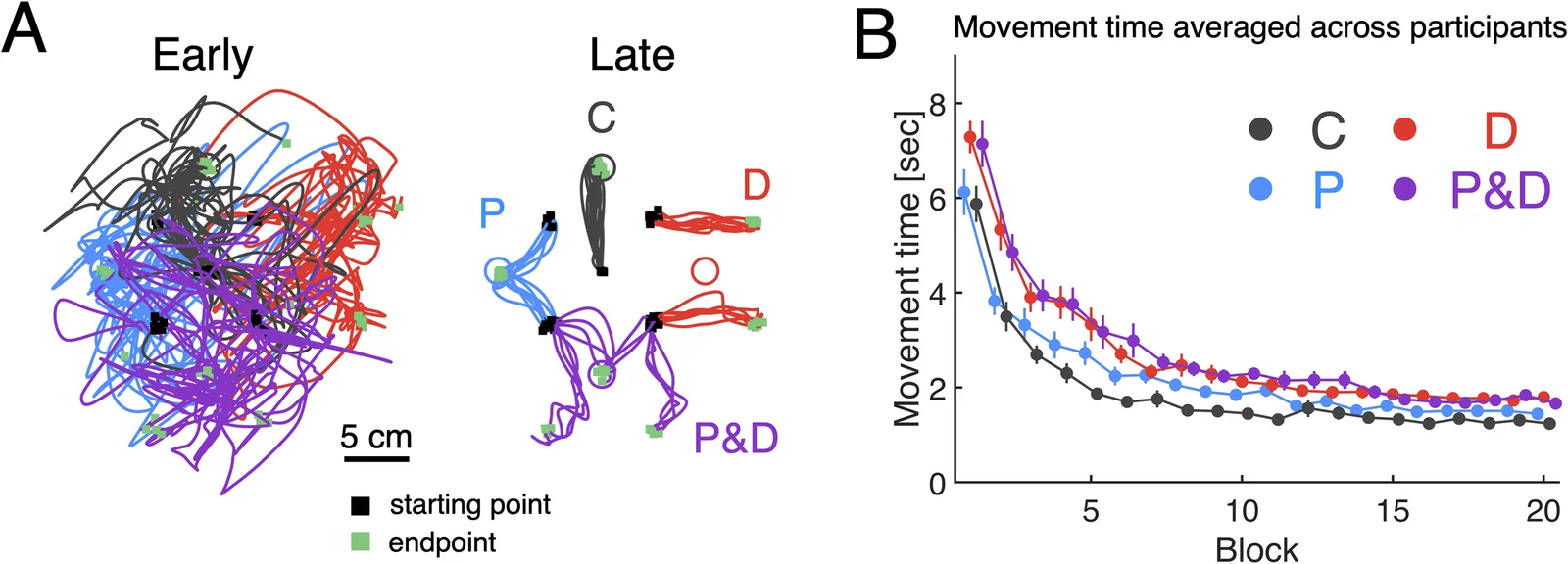
Raw cursor trajectories and learning curve. ***A***, The raw cursor trajectories of a representative participant during training trials in the early stages (first 15 trials for each condition) and the late stages (last 15 trials for each condition). The four colors represent the differences in experimental conditions (black: Control, blue: Position, red: Direction, and purple: Position and Direction). For each condition, the final trial of each sequence is displayed. The black square and green square represent the starting point and the endpoint of the cursor trajectory, respectively. ***B***, Movement time averaged across participants. Solid lines and filled circles indicate the mean across participants. Error bars indicate mean ± standard error across participants.

#### Generalization in the test trials

During the course of training, participants performed test trials on eight targets. Among the eight targets, four targets were assigned to four different conditions. Each condition was different in terms of the experience of movement direction and target position. To assess the ability to reach the target in the test trials, we characterized each reaching movement by separating it into two continuous phases: movement initiation and movement termination. The accuracy of these phases was quantified by the initial direction error and the final position error, respectively. Through training, both errors in the test trials decreased across trials (Fig. 4*A*,*D*). Because the test trials were performed without online visual feedback, this reduction reflects improvements in feedforward control. Notably, despite the lack of direct training, error reduction was observed in the P, D, and P&D conditions, indicating that generalization had occurred (Fig. 4*A*,*D*).

**Figure 4.**
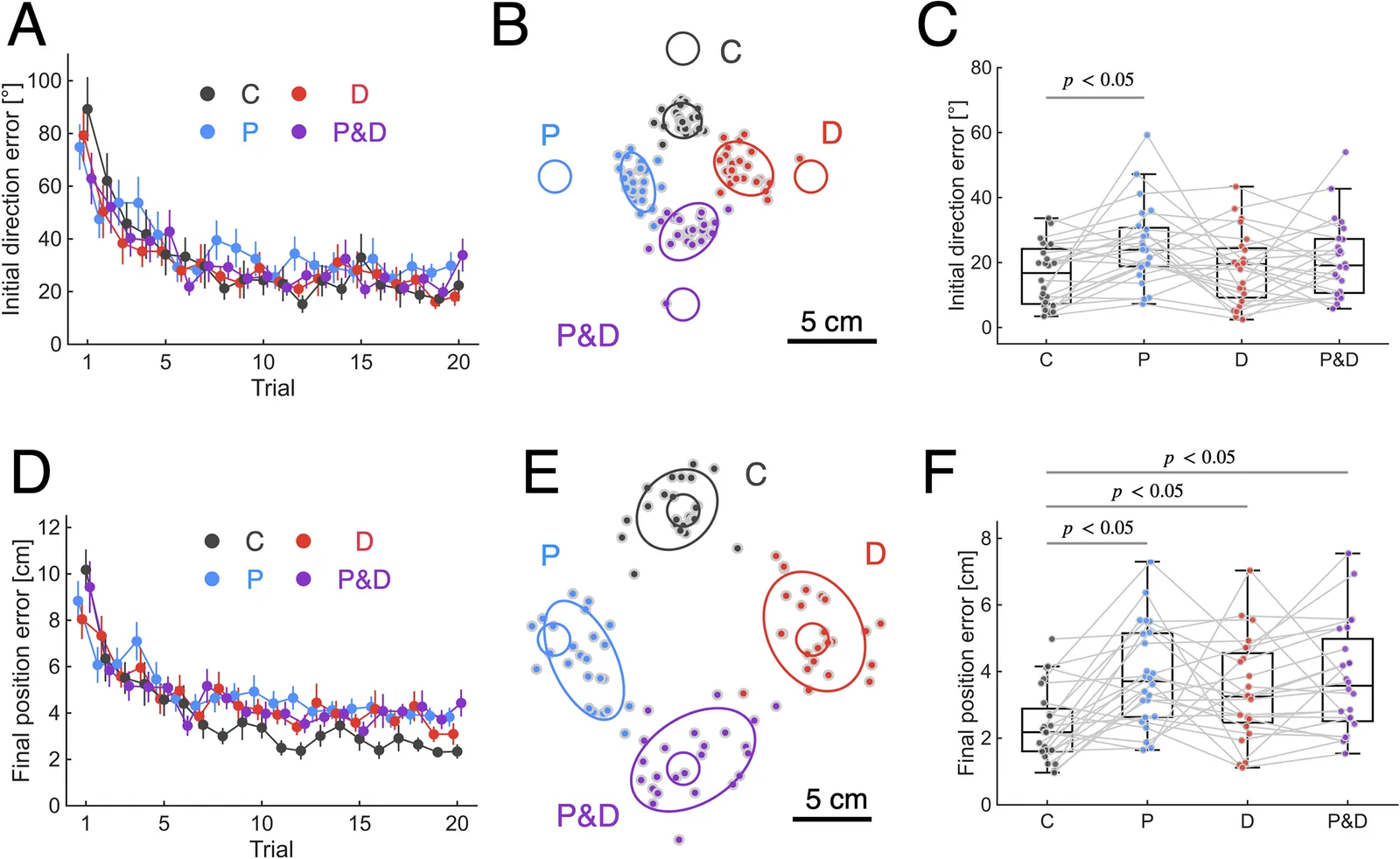
Initial direction error and final position error. ***A***, Initial direction error across test trials. Thick lines indicate the mean across participants. Error bars indicate mean ± standard error across participants. ***B***, Distribution of cursor positions at movement initiation. The dots represent the mean cursor position of the last 5 test trials for each of the 24 participants. Ellipses indicate 68% confidence intervals. ***C***, The boxplot of initial direction error. The individual dots connected by lines represent the mean error of the last 5 test trials for each of the 24 participants. ***D***, Final position error across test trials. Thick lines indicate the mean across participants. Error bars indicate mean ± standard error across participants. ***E***, Distribution of cursor positions at movement termination. The dots represent the mean cursor position of the last 5 test trials for each of the 24 participants. Ellipses indicate 68% confidence intervals. ***F***, The boxplot of final position error. The individual dots connected by lines represent the mean error of the last 5 test trials for each of the 24 participants.

**Figure 5.**
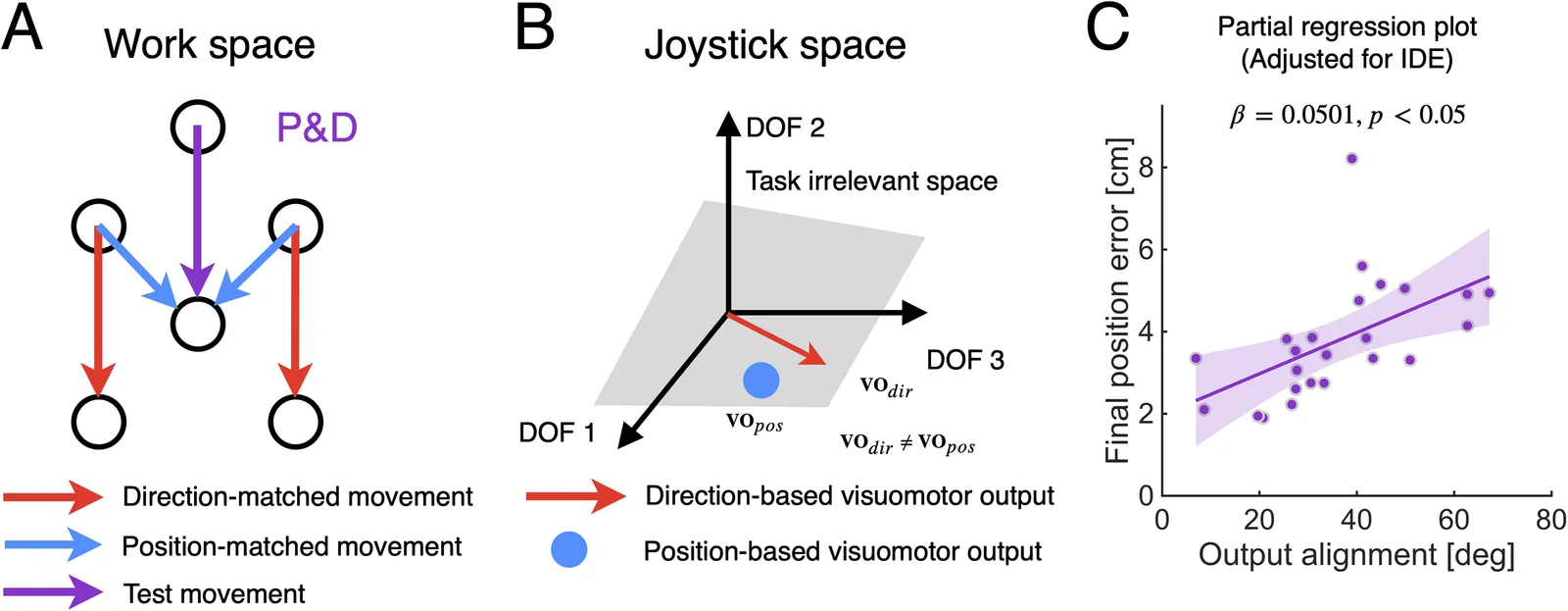
Alignment of two visuomotor outputs in the joystick control space. ***A***, The purple arrow indicates the test movement in the Position and Direction (P&D) condition. During training, participants practiced the red movement (direction-matched) and the blue movement (position-matched). The red and purple arrows share the same movement direction, while the blue and purple arrows share the same end position. Thus, in the workspace, it appears as if the direction experience (red) and position experience (blue) could be additively combined to produce the test movement (purple). ***B***, Task-irrelevant dimensions exist in the joystick control space (gray plane). As a result, the direction-based motor plan **VO**_dir_ and the position-based visuomotor outputs **VO**_pos_ do not necessarily coincide (**VO**_dir_ ≠ **VO**_pos_). The figure illustrates that these two visuomotor outputs are misaligned in the control space, emphasizing that a simple vector addition of direction and position experiences does not necessarily reproduce the test movement. ***C***, The partial regression plot shows the relationship between output alignment (*x*-axis) and final position error in the P&D condition (*y*-axis), adjusted for the initial direction error. Each dot represents an individual participant, the solid line indicates the regression line, and the shaded area denotes its 95% confidence interval.

**Figure 6.**
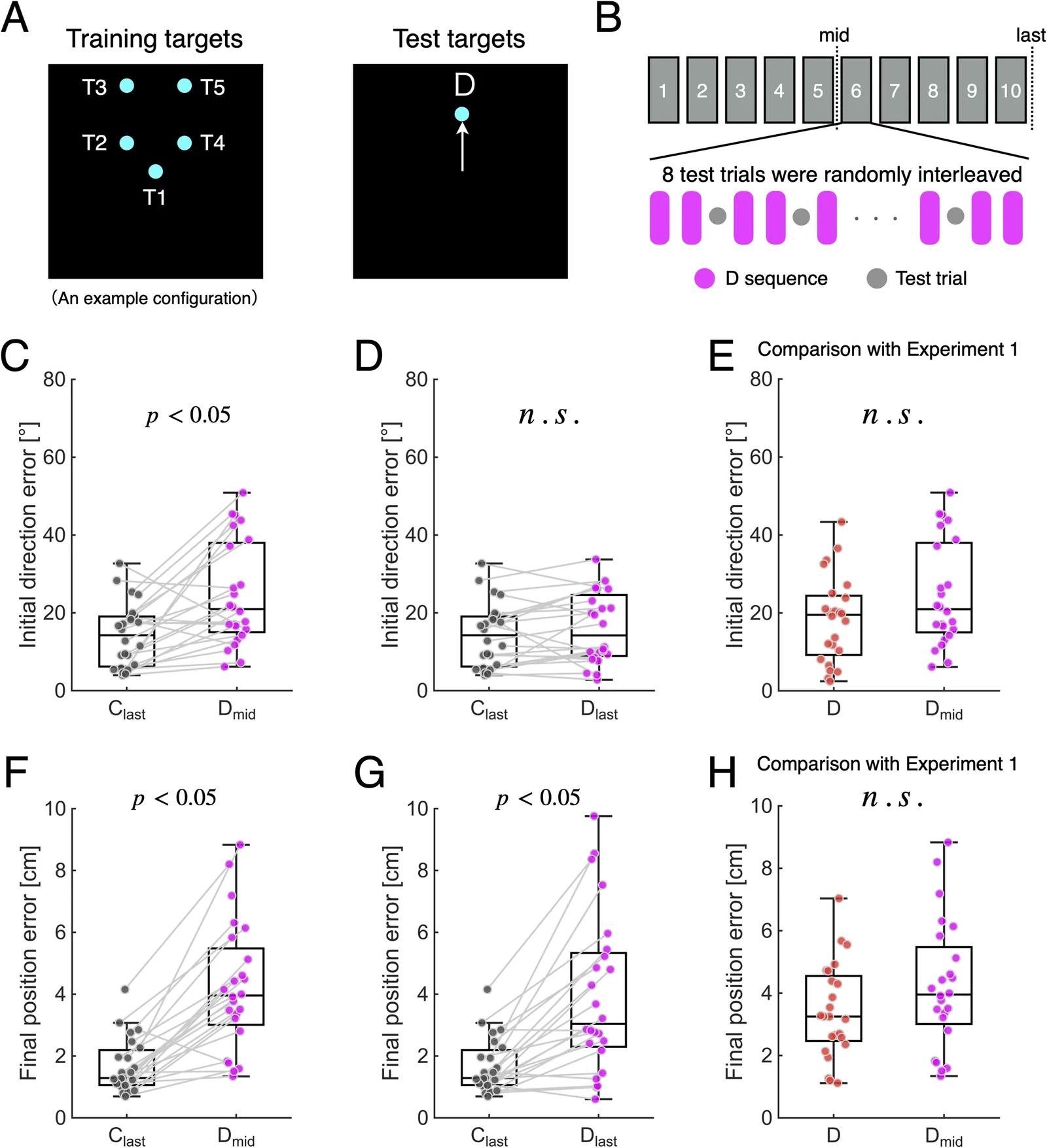
***A***, The training targets consisted of five targets, labeled T1–T5 (left). The test target was located in the direction matching the trained movements (T2 → T3 and T4 → T5; right). The assignment of the D condition to one of the four cardinal test directions was counterbalanced across participants; the configuration shown here is one example. ***B***, The task consisted of 10 blocks. In each block, there were 20 D sequences (magenta rounded rectangle) and 8 test trials (gray circle) randomly interleaved between sequences. ***C***, Boxplot of the initial direction error within Experiment 2, comparing the directly trained condition (C_last_) and the mid-training D condition (D_mid_). The individual dots connected by lines represent each participant’s mean error. ***D***, Boxplot of the initial direction error within Experiment 2, comparing C_last_ and the last-training D condition (D_last_). ***E***, Boxplot of the initial direction error in the D condition, comparing Experiment 1 (D condition) and Experiment 2 (D_mid_). Each dot represents one participant’s mean error. ***F***, Boxplot of the final position error within Experiment 2, comparing C_last_ and D_mid_. ***G***, Boxplot of the final position error within Experiment 2, comparing C_last_ and D_last_. In ***F*** and ***G***, all five test trials of one participant in C_last_ were removed as outliers, leaving no C_last_ value. Therefore, the D_mid_ (in ***F***) and D_last_ (in ***G***) data of this participant are plotted without a connecting line. ***H***, Boxplot of the final position error in the D condition, comparing Experiment 1 (D condition) and Experiment 2 (D_mid_). Each dot represents one participant’s mean error.

#### Generalization at movement initiation

Although generalization occurred in untrained conditions, the pattern of errors varied depending on the condition. We first examined the initial movement trajectory in the test trials. Figure 4*B* shows the distribution of the cursor position when the joystick velocity reached its peak. In particular, the distributions differed in shape between the P and D conditions. In the D condition, the distribution was elongated along the target direction, whereas in the P condition, it was elongated perpendicular to the target direction. This suggests that movement initiation in the D condition was aligned with the target direction. These observations were supported by a quantitative comparison of the initial direction error across conditions (Fig. 4*C*). Statistical analysis on the last 5 test trials showed that there was no significant effect of trial, indicating that the learning reached plateau [ $F_{\left(1,370.76\right)}^{a}=0.570$, *p* = 0.451], whereas there was a significant main effect of condition [ $F_{\left(3,23.08\right)}^{b}=3.98$, *p* = 0.0201]. Post hoc pairwise comparisons revealed that the lack of direction experience markedly impaired movement initiation: the initial direction error in the P condition was substantially larger than in the C condition (C − P: $t_{\left(23\right)}^{c}=-3.43$, *p* = 0.0136, *d* = −0.862, 95% CI [−16.9, −1.46]). In contrast, the lack of position experience had little effect on movement initiation. The initial direction error in the D condition was comparable to that in the C condition (C − D: $t_{\left(23\right)}^{d}=-0.861$, *p* = 0.398, *d* = −0.201, 95% CI [−9.33, 5.04]), and although the P&D condition showed a slightly larger initial direction error than the C condition (C − P&D: $t_{\left(23\right)}^{e}=-1.83$, *p* = 0.162, *d* = −0.472, 95% CI [−13.0, 2.91]), this difference did not reach statistical significance. Adding position experience to direction experience produced only a small change between the D and P&D conditions (D − P&D: $t_{\left(23\right)}^{\;f}=-1.09$, *p* = 0.346, *d* = −0.270, 95% CI [−10.5, 4.77]). Consistent with these patterns, the comparison between the P and D conditions showed a medium effect size in favor of the D condition, although the difference did not reach statistical significance (P − D: $t_{\left(23\right)}^{g}=2.37$, *p* = 0.0790, *d* = 0.661, 95% CI [−1.52, 15.6]). Altogether, these results suggest that the experience of movement direction contributed to improved accuracy of movement initiation, while the experience of target position had little effect on it.

#### Generalization at movement termination

To evaluate generalization at the termination phase under comparable initial conditions, we focused on the D and P&D conditions, in which the initial direction error was not significantly different from that in the C condition. We further included the initial direction error as a covariate in the statistical model so that the contribution of each condition to movement termination could be evaluated beyond what was already explained by movement initiation. The P condition was excluded from this focused comparison because its initial direction error was substantially larger than that of the C condition, making the termination comparison difficult to interpret under matched initial conditions. Figure 4*E* shows the distribution of the final cursor position across all four conditions. In the D condition, although the distribution was centered on the target, frequent overshooting occurred. In the P&D condition, the distribution was similarly distributed around the target with frequent deviation. Statistical analysis on the last 5 test trials showed no significant effect of trial, indicating that the learning converged [ $F_{\left(1,365.31\right)}^{h}=0.172$, *p* = 0.679], whereas there was a significant main effect of condition [ $F_{\left(3,23.28\right)}^{i}=7.98$, *p* = 7.81 × 10^−4^]. The covariate, initial direction error, also showed a strong effect [ $F_{\left(1,436.52\right)}^{\;j}=82.97$, *p* < 2.2 × 10^−16^], confirming that initial direction error was tightly linked to final position error. Post hoc pairwise comparisons revealed that, even when initial direction error was statistically controlled, the final position error in the D condition was substantially larger than in the C condition (C − D: $t_{\left(23.0\right)}^{k}=-3.09$, *p* = 0.0106, *d* = −0.850, 95% CI [−1.86, −0.064]). This indicates that, although the D condition showed comparable movement initiation to the C condition, its movement termination was less accurate. The lack of position experience thus appears to be relevant to the accuracy of movement termination, even when the movement starts from a comparable initial direction. Notably, providing position experience separately, in addition to direction experience, was not sufficient to support accurate movement termination. The final position error in the P&D condition was also significantly larger than in the C condition (C − P&D: $t_{\left(23.2\right)}^{l}=-4.18$, *p* = 0.0021, *d* = −0.988, 95% CI [−1.89, −0.347]), and was comparable to that in the D condition (D − P&D: $t_{\left(23.0\right)}^{m}=-0.529$, *p* = 0.879, *d* = −0.138, 95% CI [−1.01, 0.696]). These results indicate that, even after experiencing both target position and movement direction in separate training contexts, participants were unable to reach the level of termination accuracy achieved through direct training. What might explain this lack of additive benefit?

#### Use of the redundant control space relates to generalization at movement termination

To examine why the additive benefit was not observed at the group level, we focused on the redundancy of the joystick control space. Although position and direction experiences appear additive in the workspace, the same target position can be achieved by infinitely many joystick configurations along the task-irrelevant dimensions (Fig. 5*A*,*B*). Consequently, the visuomotor outputs derived from direction and position experiences do not necessarily coincide. We therefore quantified the alignment between the direction-based and position-based visuomotor outputs in the joystick control space, defined as the angle between the two outputs. To examine whether this alignment explains individual differences in the final position error in the P&D condition, we performed a linear regression at the participant level with the alignment and initial direction error as predictors of the final position error. After controlling for initial direction error, the alignment was a significant predictor of the final position error (*β*^*n*^ = 0.0501, *p* = 0.00692; Fig. 5*C*), indicating that participants with greater alignment exhibited smaller final position errors in the P&D condition. These results indicate that the alignment between direction- and position-based outputs in the redundant control space underlies how effectively position experience and direction experience are combined to support accurate movement termination.

### Experiment 2

#### Generalization in the D condition under single-condition training

The D condition provides the central evidence for our main findings—that directional experience contributes to movement initiation, whereas the lack of positional experience is associated with reduced accuracy of movement termination. To examine whether interleaving the four training conditions in Experiment 1 interfered with the generalization observed in the D condition, we trained a separate group of participants exclusively on the D (Direction) sequence. C_last_ and D_mid_ were matched to the C and D conditions of Experiment 1 in the amount of practice.

Results showed the initial direction error in D_mid_ was larger than in C_last_ (C_last_ − D_mid_: $t_{\left(23\right)}^{o}=-4.05$, *p* = 0.0005, *d* = −0.99, 95% CI [−16.0, −5.16]; Fig. 6*C*). Thus, at this matched-practice point, Experiment 2 did not yet reproduce the comparability between the D and C conditions that had been observed in Experiment 1. However, with continued training the initial direction error in D_last_ converged to a level comparable to C_last_ (C_last_ − D_last_: $t_{\left(22.9\right)}^{\;p}=-1.49$, *p* = 0.150, *d* = −0.21, 95% CI [−4.71, 0.77]; Fig. 6*D*), with an effect size closely matching that of the C − D comparison in Experiment 1 (*d* = −0.201). The comparable movement initiation between the D and C conditions observed in Experiment 1 was therefore reproduced after additional training. A direct comparison of the initial direction error in the D condition between the two experiments revealed no significant difference, although the error tended to be smaller in Experiment 1 (D − D_mid_: $t_{\left(46\right)}^{q}=-1.80$, *p* = 0.079, *d* = −0.60, 95% CI [−13.6, 0.77]; Fig. 6*E*), suggesting that interleaving the four conditions in Experiment 1 may have influenced the rate of learning. Together, these results indicate that, although the within participants design in Experiment 1 may have accelerated the decrease of the initial direction error, directional experience alone was ultimately sufficient to achieve movement initiation comparable to the C condition, reproducing the corresponding finding of Experiment 1.

A similar pattern was observed for the final position error. The final position error in D_mid_ was larger than in C_last_ (C_last_ − D_mid_: $t_{\left(25.9\right)}^{r}=-6.27$, *p* < 0.0001, *d* = −1.63, 95% CI [−2.70, −1.37]; Fig. 6*F*), reproducing the impaired movement termination under directional experience alone observed in Experiment 1. This error remained larger than in C_last_ even with continued training (C_last_ − D_last_: $t_{\left(23.1\right)}^{s}=-5.09$, *p* < 0.0001, *d* = −2.09, 95% CI [−3.02, −1.27]; Fig. 6*G*), indicating that additional practice did not improve movement termination. As with the initial direction error, a direct comparison between the two experiments revealed no significant difference (D − D_mid_: $t_{\left(46.6\right)}^{t}=-0.901$, *p* = 0.372, *d* = −0.301, 95% CI [−1.4, 0.532]; Fig. 6*H*).

In summary, these results confirm that the central dissociation of Experiment 1 is not attributable to the interleaving of the four conditions: even with training on the D sequence alone, directional experience was sufficient to learn accurate movement initiation but not accurate movement termination, reproducing the central claim of Experiment 1. At the same time, we acknowledge that interleaving the four conditions did affect the learning of the D condition in Experiment 1, indicating that some interference was present, possibly because participants spent more total time engaged in the task across the interleaved conditions.

## Discussion

This study aimed to clarify how two types of information, movement direction and target position, contribute to de novo motor learning. To this end, we adopted a generalization paradigm to dissociate the respective contributions of direction and position experiences (Malfait et al., 2002; Donchin et al., 2003; Shadmehr, 2004). The results suggested that direction experience enhanced the accuracy of movement initiation, whereas the absence of position experience led to reduced accuracy of movement termination (Fig. 4). However, experiencing position and direction separately did not yield additive effects at the group level (Fig. 4*F*). Instead, participants whose position- and direction-based visuomotor outputs were more closely aligned in the redundant control space exhibited smaller final position errors (Fig. 5*C*).

### Contribution of direction experience

Our results demonstrate that directional experience selectively enhances the accuracy of movement initiation (Fig. 4*C*), supporting the long-standing view that motor learning primarily relies on direction-based representations. This view is supported by classical findings in motor adaptation showing that adaptation to visuomotor rotations involves the remapping of movement vectors rather than final limb positions (Wang and Sainburg, 2005). Evidence from de novo motor learning studies further supports this direction-based representation. When participants acquired novel hand-to-cursor mappings, their trajectories became progressively straighter with practice, indicating the establishment of a direction-based transport map linking changes in hand posture to cursor motion (Mosier et al., 2005). Moreover, Ranganathan et al. (2013) demonstrated that the motor system exploits redundancy not by converging to a single final posture, but by adopting distinct hand postures for a single target location, depending on the starting hand configuration. This dependence on the initial state reinforces the dominance of a transport map, i.e., associating cursor displacement with changes in hand posture (Ranganathan et al., 2013). Consistent with these studies, our results showed that isolated experience of movement direction improved the accuracy of movement initiation to a level comparable to that achieved through direct practice (Fig. 4*C*). Altogether, these findings suggest that directional experience contributes to the refinement of direction-based control mechanisms that support accurate movement initiation.

### Contribution of position experience

Although initial direction error in the Direction (D) condition was comparable to the Control (C) condition, the final position error was significantly larger in the D condition, indicating impaired accuracy in movement termination (Fig. 4). This dissociation between movement initiation and termination can be explained by the existence of two complementary control processes: a transient movement controller that generates vectorial commands for reaching, and a sustained hold controller that stabilizes the limb at the desired endpoint (Shadmehr, 2017; Albert et al., 2020). Evidence for this distinction comes from multiple lines of research. In adaptation studies, learning acquired in visuomotor rotation tasks generalizes poorly between trajectory-reversal (slicing) and positioning (reaching) tasks, suggesting that initial trajectory and final position rely on separate neural mechanisms (Ghez et al., 2007; Scheidt and Ghez, 2007). Neurophysiological data further indicate that neurons in the primary motor cortex exhibit separate load-related tuning during posture and movement, reinforcing the functional separation between control phases (Kurtzer et al., 2005). Within this framework, the impaired termination observed in the D condition is consistent with insufficient formation of the hold controller, which appears to rely on positional experience. Although the D condition supported accurate movement initiation, the lack of positional experience was associated with reduced accuracy at movement termination. This pattern suggests that positional experience plays a role in the formation of the hold controller, which becomes relevant after movement initiation has been guided by directional experience.

### Conditional contribution of position experience to movement termination

The previous section suggested that position experience plays a role in supporting accurate movement termination. If this were the case, providing position experience in addition to direction experience (as in the P&D condition) would be expected to improve movement termination compared with direction experience alone (D condition). However, additive benefits of providing both experiences were not observed at the group level. This is likely because, in the P&D condition, the visuomotor outputs derived from direction and position experiences do not necessarily coincide due to the redundancy in the control space. In support of this view, participants with higher alignment between direction- and position-based visuomotor outputs in the joystick control space exhibited smaller final position errors (Fig. 5*C*). This finding suggests that, even when both direction and position experiences are acquired, the benefit of position experience emerges only when the position-based and direction-based visuomotor maps are aligned with each other in the redundant control space. This account also fits within the framework of movement and hold controllers (Shadmehr, 2017; Albert et al., 2020), in which the alignment between the two visuomotor maps may reflect the agreement between the states specified by these controllers. Taken together, these results suggest that position experience does not uniformly support movement termination, but its effect is conditional—specifically when it is integrated with direction experience within a coherent visuomotor representation.

### Distinction between de novo motor learning and motor adaptation

De novo motor learning fundamentally differs from motor adaptation in that it necessitates establishing entirely new mappings between motor commands and sensory outcomes, rather than merely recalibrating pre-existing ones. Although prior motor adaptation studies have revealed the existence of separate movement and hold controllers (Ghez et al., 2007; Scheidt and Ghez, 2007), the specific sensorimotor experiences required for their initial construction remain largely unexplored within that traditional paradigm. By utilizing de novo motor learning, we were able to precisely control the available experiences, revealing that different types of experience support the acquisition of these two distinct controllers. A second key distinction lies in the treatment of redundancy. Motor adaptation typically involves recalibration within a constrained control space (Berger et al., 2013; Sadtler et al., 2014; Berger et al., 2022), whereas de novo motor learning requires the exploration of solutions in an unconstrained high-dimensional space (Mosier et al., 2005; Choi et al., 2020; Dal’Bello and Izawa, 2021). This fundamental difference introduces a distinct computational challenge. When direction- and position-based visuomotor maps are constructed separately in a high-dimensional space, the motor commands specified by each map are not guaranteed to be mutually consistent. As a result, the state reached by the direction-based map may not coincide with the state stabilized by the position-based map, leading to ineffective movement termination. Consequently, in de novo motor learning, achieving coordination between these newly acquired visuomotor maps emerges as a critical computational requirement for accurate reaching movements. From the perspective of ecological dynamics (Gibson, 1979; Jacobs and Michaels, 2007), this distinction can also be framed in terms of the formation of the task-specific perception–action couplings. Whereas motor adaptation can be viewed as the recalibration of pre-existing couplings under novel task constraints, de novo motor learning requires learners to establish entirely new couplings between their actions and visual consequences. The present findings suggest that the formation of these new couplings is not unitary, but involves the parallel establishment of distinct couplings: one between cursor-direction information and movement initiation-related actions, and another between cursor position information and movement termination-related actions.

### Neural representations underlying direction- and position-based learning

Direction- and position-based learning may rely on distinct representations of the target, such as a cursor-centered displacement vector specifying movement direction and an absolute target position anchored in external space. These two forms of information are broadly consistent with the distinction between allocentric and egocentric spatial representations (Schenk, 2006; Milner and Goodale, 2008; Chen and Crawford, 2020). From this perspective, the two forms of learning observed in our study can be interpreted in relation to these spatial codes: position-based learning may reflect reliance on egocentric representations of target location, whereas direction-based learning may reflect reliance on allocentric representations of the spatial relation between the cursor and the target. This interpretation is consistent with the idea that multiple spatial representations can be maintained in parallel to support flexible visuomotor behavior.

### Conclusion

In conclusion, our results show that de novo motor learning relies on both movement direction and target position experiences. Direction experience determined the accuracy of movement initiation, whereas the accuracy of movement termination depended on the alignment between direction- and position-based outputs in the redundant control space. These findings suggest that accurate formation of novel sensorimotor maps requires the coordinated use of directional and positional experiences.

Our data revealed that this coordination varied considerably across individuals and was associated with endpoint accuracy. Building on this observation, future work will examine the origin of these individual differences and whether such coordination can be actively trained through targeted practice. Investigating these questions will deepen our understanding of how the brain integrates multiple types of sensorimotor experience to construct novel sensorimotor maps.

## Synthesis

Reviewing Editor: Gergely Silasi, University of Ottawa Decisions are customarily a result of the Reviewing Editor and the peer reviewers coming together and discussing their recommendations until a consensus is reached. When revisions are invited, a fact-based synthesis statement explaining their decision and outlining what is needed to prepare a revision will be listed below. The following reviewer(s) agreed to reveal their identity: cameron mang, Rajiv Ranganathan.

As you can see from the comments below both reviewers provided several suggestions for improving your manuscript. Reviewer 2 raised several concerns that need to be addressed and they are listed in order of priority (first 2 being most essential). Specifically, for point #1 regarding the independent contributions of position and direction, additional comparisons were suggested. In addition, independent analyses of movement extent was also suggested to clarify any potential differences between conditions. The second point could be addressed by additional experiments as suggested by the reviewer, or by any potential evidence within the current dataset showing that participants can learn the 4 positions independently without influencing each other.

Reviewer 1:

Overview: The manuscript describes a clever and well-thought out experiment designed to study a classic problem in human motor control. The manuscript is organized and presents complex information in an understandable way. Addressing comments below may help to strengthen the rationale for the work and improve reader understanding of the methods used.

Introduction:

The example used to characterize the importance of the topic area could be improved. For instance, is it accurate to suggest that learners have 'no idea' of how controller inputs translate to on-screen movement when playing a new video game? In most cases, there are conventional controls that learners have experienced previously and may need to adapt. Furthermore, it could be argued that after developmental phases of growth and maturation, there are relatively few instances in which individuals engage in truly de novo motor learning. Perhaps an individuals first experience with controlling an external effector is more salient (e.g., remote control application, driving).

The wording of the hypothesis and the distinction between "separate" and "independent" or "alone" could be clarified. "We hypothesized that if vector and position coding additively contribute to the acquisition of a de novo sensorimotor map, learning both components separately would lead to greater generalization than learning either component alone." The distinction is difficult to interpret as written here and later in the manuscript. Additionally, more explanation is needed to convince the reader that learning these components separately might be beneficial and why this is important.

Materials and Methods:

Please explain the inclusion of only right-handed individuals for a bi-manual task.

Please provide more information regarding the PCA performed on the calibration data. Include a brief explanation of what the components derived from a PCA represent.

Line 137 - Why is it described as "every few" trials - was there a specific number of trials that could be reported?

Line 146 - Please provide a brief explanation of what the 'score' reflects.

Line 169 - The feedback message of "Stop a little earlier" strikes me as problematic. Could a learner interpret this as meaning that they are reaching beyond the target and need to "stop earlier" to stop on the target? Why was this particular message used instead of something else, such as "move faster" or "too slow".

Line 243 - When considering movement direction, did the authors also consider using a measure of path length or cumulative distance? Please explain why only the initial direction error is relevant if mapping a movement trajectory.

Line 276 - Please add information to the outlier identification section. Specifically, was this procedure completed on group data or individual data?

In Figure 3B, the representative data seems to suggest that the participant moved faster under the position condition. Was this a common observation?

Results:

Generally, the results are presented clearly and comprehensively. One possible addition would be the movement time data for the full data set rather than just the representative data shown in Figure 3B.

In the Figure 3 caption, it is stated that: "Thick lines indicate the mean across participants." It is not clear what thick lines are being referenced to in this statement.

Discussion:

As expected, the discussion is heavily focused on concepts in computational motor control. The authors may consider briefly acknowledging concepts in ecological dynamics, such as direct perception and perception-action coupling, when interpreting their findings in the sub-section on 'Distinction between de novo motor learning and motor adaptation'.

Consider adding information to the conclusion that describes the logical next step of this work.

Reviewer 2:

This is a review of "Distinct roles of directional and positional experience in de novo visuomotor learning"

The main question that the authors pose is what types of sensorimotor information contribute to denovo learning. Using a novel joystick to cursor task, the authors employed a generalization task to restrict learning to one of three different conditions - (i) target position, (ii) movement direction, and (iii) both components. The authors claim that direction experience improved movement initiation, whereas position experience enhanced movement termination.

Although the question posed by the authors is intriguing, I have several major concerns in its current form:

1. Evidence for "independent" contributions of position and direction: The evidence for the main claim is somewhat ambiguous - clearly the C condition performance is the best in all conditions, and the authors make conclusions depending on how much "worse" other conditions are relative to this condition. However, there is no direct comparison of other conditions to each other. For example, the authors only show a "weak trend" (p = .08) that direction experience had smaller initial direction error than the position condition - an important prediction that is not fully supported by the evidence

Similarly, in no analysis is the P condition "better" than the D condition - i.e., the authors argue for position as contributing to movement termination, but the final direction error is confounded by both initial direction and movement extent. An independent analysis of movement extent (instead of final position error) could provide greater information as to whether learning position helped participants learn movement extent (independent of variability in movement direction) - i.e., where the P condition would be better than the D condition

2. Repeated-measures experimental design: Another major concern is the experimental design: the authors examine learning here using a generalization paradigm with fully repeated measures where the same participant experiences all 4 conditions in different parts of the workspace. For 'denovo' learning, where participants are learning from scratch, this design seems a bit unconventional because it assumes that participants can learn the 4 directions (which are interleaved during training and have different information) independently and simultaneously. At the very least, this should be followed up by a "between-group" design to show that there is no "interference" between learning one condition vs. learning 4 conditions.

Relatedly, this experimental design assumes that all parts of the workspace are "equal" - however the PC analysis typically has an "easier' PC1 relative to PC2 (depending on the relative variances accounted by the PCs) - in other words, control along the X and Y axes may not be 'equal' in this task and there may also be biomechanical factors that drive the relative ease of the different directions (Fig 3 hints at some of this, where the movement time is not the same across all 4 conditions even initially in learning; but no statistics are provided here). The PC data could be helpful in assessing it. Alternatively, a control experiment where the positions of the P, C, D and P&D altered could be used to show that these effects are not artifacts of biomechanical constraints or the PCs

3. Redundancy analyses - the authors clearly use a redundant task here - yet, no analysis of the redundancy is performed (especially to check if the use of the redundant space changed with condition). This could provide more insight into the controller rather than simply looking at task performance, and could bolster their final conclusion of the correlations in the P&D condition

Other:

At several points, the authors indicate that there was "No effect of trial, indicating learning has plateaued". This statement is not clear given the clear learning curve presented in Fig 4 that shows that errors decrease with trials. Please clarify in the Results that only the last 5 trials were used for analysis.

Please indicate if the joysticks are spring-loaded (i.e., they return to the center when the fingers are removed)

Please provide some indication of what the PC loadings looked like across participants, especially if the mappings generally resulted in "independent' hand controls (i.e. one joystick controls X, the other controls Y etc.) or if they were typically coupled.

Figure 4 show a large number of data points on the box plots/scatter plots (even though there are only 24 participants). Within-participant data should not be displayed at the same level as between-participant data since they represent different things and can be misleading).

The inference for "Alignment" may be better captured by the angle between VOdir and VOpos (rather than the magnitude of the difference vector)

The discussion about "Neural representations" seems far too speculative to tie directly to the results here. At the very least, it can be shortened and focused on how this actually relates to the conclusions of this study

## Author Response

Response to Reviewer#1 >Overview: The manuscript describes a clever and well-thought out experiment designed to study a classic problem in human motor control. The manuscript is organized and presents complex information in an understandable way. Addressing comments below may help to strengthen the rationale for the work and improve reader understanding of the methods used.

We would like to thank the reviewer for the invaluable comments that were helpful. We have responded to all the points raised and revised the manuscript accordingly. Also, the revised sentences following the reviewer#1's indications and comments in the revised manuscript were highlighted in yellow.

Introduction >The example used to characterize the importance of the topic area could be improved. For instance, is it accurate to suggest that learners have 'no idea' of how controller inputs translate to on-screen movement when playing a new video game? In most cases, there are conventional controls that learners have experienced previously and may need to adapt. Furthermore, it could be argued that after developmental phases of growth and maturation, there are relatively few instances in which individuals engage in truly de novo motor learning. Perhaps an individuals first experience with controlling an external effector is more salient (e.g., remote control application, driving).

We thank the reviewer for this insightful comment. We completely agree that our previous example of playing a new video game was not entirely accurate. As you pointed out, players often rely on prior experience with conventional controls, making it more akin to motor adaptation rather than true de novo learning. We also agree that an individual's first experience controlling an external effector serves as a much more salient and appropriate example of de novo motor learning. Following your excellent suggestion, we have revised the Introduction to use the example of operating a remote controlled device (such as a drone or robotic manipulator). Furthermore, we have removed the exaggerated phrase "no idea" and replaced it with a more precise description stating that individuals "must learn" the novel translation of motor commands.

Changes in the manuscript (Introduction, p. 2, ll. 36-39): ...This capability underlies many everyday skills, such as playing musical instruments or using unfamiliar tools. For example, when first operating a remote-controlled device (e.g., a drone or robotic manipulator), individuals must learn how their motor commands applied to a handheld device translate into movements of a spatially separate external effector. De novo motor learning refers to the process of establishing this novel relationship... >The wording of the hypothesis and the distinction between "separate" and "independent" or "alone" could be clarified. "We hypothesized that if vector and position coding additively contribute to the acquisition of a de novo sensorimotor map, learning both components separately would lead to greater generalization than learning either component alone." The distinction is difficult to interpret as written here and later in the manuscript. Additionally, more explanation is needed to convince the reader that learning these components separately might be beneficial and why this is important.

We thank the reviewer for this constructive feedback. We completely agree that the distinction between these terms was confusing, and that the rationale for our experimental design needed more elaboration. (1) Clarification of terminology. We have explicitly defined "separately" and "alone" in the Introduction, and have unified our terminology throughout the manuscript by replacing ambiguous words such as "independently" with "alone" in the Results section. (2) Rationale for learning components separately. We have added an explanation of why this design is informative. Specifically, this design tests whether the sensorimotor system can integrate separate learning experiences. We reasoned that if direction and position information additively contribute to map formation, then acquiring both components should yield greater generalization than experiencing either component alone.

Changes in the manuscript (Introduction, p. 3, ll. 78-88): ... generalizes to untrained movements. In the experiment, three distinct target conditions were designed to elicit generalization from experience with specific components: one target emphasized target position alone, another emphasized movement direction alone, and a third encouraged learning of both elements separately. We define "separately" as experiencing both the matched position and direction of an untrained movement through distinct training contexts, whereas "alone" refers to experiencing only one of these matched components. This experimental design is crucial for examining whether the sensorimotor system can integrate separate learning experiences. We reasoned that if direction and position information additively contribute to the construction of a sensorimotor map, then acquiring both components should lead to greater generalization than experiencing either component alone.

Materials and Methods: >Please explain the inclusion of only right-handed individuals for a bi-manual task.

We restricted participation to right-handed individuals to control for variability associated with handedness. Because the aim of this study was not to examine differences related to handedness, we limited the sample to right-handed participants to maintain a more homogeneous participant group and reduce potential confounds. >Please provide more information regarding the PCA performed on the calibration data. Include a brief explanation of what the components derived from a PCA represent.

Thank you for this suggestion. We have added a brief explanation of PCA to the Calibration task section of the Methods. Specifically, we clarified that PCA identifies orthogonal directions in the data that successively capture the largest variance, such that each principal component represents a dominant pattern of coordinated joystick movements.

Changes in the manuscript (Methods, p. 5, ll. 129-132): ... contributed to the mapping matrix A. Principal component analysis (PCA) was then performed on the standardized calibration data. PCA identifies orthogonal directions in the data that successively capture the largest variance, such that each principal component represents a dominant pattern of coordinated joystick movements. >Line 137 - Why is it described as "every few" trials - was there a specific number of trials that could be reported? Thank you for pointing this out. We have revised the text to specify the exact number of trials. The number of trials in each sequence was two or three, depending on the condition (see the Experimental conditions section for details).

Changes in the manuscript (Methods, p. 6, ll. 147-152):

At the end of every 2 or 3 training trials (referred to as a sequence of trials), the visual feedback of the cursor was removed and participants were instructed to relax the joysticks (i.e., release their hands so that the spring force passively returned each joystick to the center position) to reset the cursor position at the center of the workspace (Figure 2B). The number of trials in each sequence differed across experimental conditions (See Experimental conditions). >Line 146 - Please provide a brief explanation of what the 'score' reflects.

Thank you for pointing this out. We agree that a brief explanation helps clarify the purpose of the score. Accordingly, we have added a phrase to explicitly state that the score reflects the speed of the reaching movement. The text has been revised as follows:

Changes in the manuscript (Methods, p. 6, ll. 156-158):

To motivate them to follow this instruction, we provided a score calculated based on the elapsed time after the start of the trial, which reflects how quickly they reached the target. >Line 169 - The feedback message of "Stop a little earlier" strikes me as problematic. Could a learner interpret this as meaning that they are reaching beyond the target and need to "stop earlier" to stop on the target? Why was this particular message used instead of something else, such as "move faster" or "too slow".

Thank you for raising this valid concern. We understand that the phrase "stop a little earlier" could potentially be interpreted as spatial feedback. However, we believe this misinterpretation was unlikely in our experiment because the exact meaning of this message was clearly explained to the participants during the task instructions prior to the experiment. Specifically, we instructed the participants as follows: "If it takes too long from the start of the trial until the stick stops moving, a warning message in red text saying "Please stop more quickly" will be displayed at the end of the trial.

To avoid this warning, stop the stick's movement in a single motion." Through these instructions, participants clearly understood that the warning was related to the temporal duration of their movement rather than spatial accuracy. To address your concern and clarify this for the readers, we have added a brief explanation to the manuscript detailing that participants were explicitly instructed about the temporal nature of this warning.

Changes in the manuscript (Methods, p. 7, ll. 181-185):

If the time between the start of the trial and the offset exceeded 2 seconds, participants were provided with a warning message, "stop a little earlier". To prevent participants from misinterpreting this message as spatial feedback (e.g., stopping short of the target), we explicitly instructed them beforehand that this warning indicated the movement duration was too long, and we encouraged them to complete their movement in a single motion. >Line 243 - When considering movement direction, did the authors also consider using a measure of path length or cumulative distance? Please explain why only the initial direction error is relevant if mapping a movement trajectory.

Thank you for this thoughtful comment. We would like to clarify the rationale for our choice of measures. Our aim was not to characterize the movement trajectory as a whole, but rather to assess how generalization manifests in different parts of the movement. Specifically, we decomposed the reaching movement into its initial phase (movement initiation) and terminal phase (movement endpoint), and evaluated generalization separately for each phase. The initial direction error captures the accuracy of the former, while the final position error captures the accuracy of the latter. Measures that summarize the entire trajectory as a single scalar value, such as path length or cumulative distance, are not suited for this purpose because they do not distinguish between the initial and terminal phases of the movement. Moreover, such measures can yield small values even when the movement is not appropriately directed toward the target, as long as the movement magnitude happens to match the target distance. For these reasons, we relied on initial direction error and final position error as complementary measures that together characterize the spatial accuracy of the reaching movement. >Line 276 - Please add information to the outlier identification section. Specifically, was this procedure completed on group data or individual data? We sincerely appreciate your careful reading and for pointing out this important detail. We have clarified this point in the revised manuscript. The outlier removal procedure was completed on the group data. Specifically, we pooled the data from all participants within each condition before applying the quartile method. We have added a sentence to the "Outlier criteria" section to explicitly state this procedure.

Changes in the manuscript (Methods, p. 11, ll. 300-301):

To avoid outlier influenced bias during hypothesis testing, outliers were removed using the quartile method. Specifically, this procedure was applied to the group data pooled across all participants within each condition. First, the first quartile (Q1) and the third quartile (Q3)... >In Figure 3B, the representative data seems to suggest that the participant moved faster under the position condition. Was this a common observation? Thank you for your comment. We apologize for the confusion. While Figure 3A shows the trajectories of a single representative participant, Figure 3B actually plots the average movement time across all participants, as described in the figure caption. Therefore, the tendency you observed-that movements under the Position condition were relatively faster in the early blocks-is indeed a common observation across the group, rather than being specific to a single individual.

To prevent this misunderstanding for future readers, we have added the title "Movement time averaged across participants" directly to Figure 3B to explicitly indicate that the plot represents the group mean.

Results: >Generally, the results are presented clearly and comprehensively. One possible addition would be the movement time data for the full data set rather than just the representative data shown in Figure 3B. In the Figure 3 caption, it is stated that: "Thick lines indicate the mean across participants." It is not clear what thick lines are being referenced to in this statement.

Thank you for your positive feedback and constructive suggestions.

Regarding your first point, we apologize for the confusion. Figure 3B actually already presents the average movement time for the full data set (all participants), not just a single representative participant. As mentioned in our response to a previous comment, to make this immediately clear to the readers, we have added the title "Movement time averaged across participants" directly to Figure 3B.

Regarding your second point, we completely agree that the phrase "thick lines" in the caption was ambiguous. It was originally intended to refer to the lines connecting the data points in Figure 3B. To eliminate this ambiguity and accurately describe the figure, we have revised the figure caption to specifically mention the solid lines and filled circles.

Changes in the manuscript (Caption in Figure 3, p. 28, ll. 773-774): ... cursor trajectory, respectively. B: Movement time averaged across participants. Solid lines and filled circles indicate the mean across participants. Error bars indicate mean standard error across participants.

Discussion: >As expected, the discussion is heavily focused on concepts in computational motor control. The authors may consider briefly acknowledging concepts in ecological dynamics, such as direct perception and perception-action coupling, when interpreting their findings in the sub-section on 'Distinction between de novo motor learning and motor adaptation'.

We thank the reviewer for this suggestion. Following the reviewer's recommendation, we have added a brief discussion of ecological dynamics perspectives at the end of the sub-section 'Distinction between de novo motor learning and motor adaptation'. Specifically, we have framed the distinction between de novo motor learning and motor adaptation in terms of perception-action coupling: whereas motor adaptation involves the recalibration of pre-existing couplings, de novo motor learning requires the formation of entirely new ones. We further suggest that this coupling formation is not a unitary process, but involves the parallel establishment of distinct couplings-between directional perceptual information and movement initiation, and between positional perceptual information and movement termination.

Changes in the manuscript (Discussion, p. 22, ll. 628-637): ... accurate reaching movements. From the perspective of ecological dynamics (Gibson, 1979; Jacobs and Michaels, 2007), this distinction can also be framed in terms of the formation of task-specific perception-action couplings. Whereas motor adaptation can be viewed as the recalibration of pre-existing couplings under novel task constraints, de novo motor learning requires learners to establish entirely new couplings between their actions and visual consequences. The present findings suggest that the formation of these new couplings is not unitary, but involves the parallel establishment of distinct couplings: one between cursor-direction information and movement initiation-related actions, and another between cursor-position information and movement termination-related actions. >Consider adding information to the conclusion that describes the logical next step of this work.

We thank the reviewer for this constructive suggestion. Following the reviewer's recommendation, we have expanded the conclusion to describe the logical next steps that emerge from our findings.

Change in manuscript (Discussion, p. 23, ll. 658-663):

Our data revealed that this coordination varied considerably across individuals and was associated with endpoint accuracy. Building on this observation, future work will examine the origin of the individual differences and whether such coordination can be actively trained through targeted practice. Investigating these questions will deepen our understanding of how the brain integrates multiple types of sensorimotor experience to construct novel sensorimotor maps.

Response to Reviewer#2 >The main question that the authors pose is what types of sensorimotor information contribute to de novo learning. Using a novel joystick to cursor task, the authors employed a generalization task to restrict learning to one of three different conditions - (i) target position, (ii) movement direction, and (iii) both components. The authors claim that direction experience improved movement initiation, whereas position experience enhanced movement termination.

We would like to thank the reviewer for the thorough and insightful comments. We have responded to all the points raised and revised the manuscript accordingly. The revised sentences following the reviewer#2ʼs indications and comments in the revised manuscript were highlighted in cyan.

Major comments >(1) Even Evidence for "independent" contributions of position and direction: The evidence for the main claim is somewhat ambiguous - clearly the C condition performance is the best in all conditions, and the authors make conclusions depending on how much "worse" other conditions are relative to this condition. However, there is no direct comparison of other conditions to each other.

For example, the authors only show a "weak trend" (p = .08) that direction experience had smaller initial direction error than the position condition - an important prediction that is not fully supported by the evidence Similarly, in no analysis is the P condition "better" than the D condition - i.e., the authors argue for position as contributing to movement termination, but the final direction error is confounded by both initial direction and movement extent. An independent analysis of movement extent (instead of final position error) could provide greater information as to whether learning position helped participants learn movement extent (independent of variability in movement direction) - i.e., where the P condition would be better than the D condition.

We sincerely thank the reviewer for this critical and constructive comment. We agree that our original claim about the independent contributions of position and direction experiences was not sufficiently supported by the data. In response, we have made three major revisions: (ⅰ) we have substantially revised our interpretation regarding position experience and no longer claim that it contributes independently of direction experience; (ⅱ) we have addressed the methodological concern about the confound between initial direction error and final position error by including the former as a covariate in the statistical model; and (ⅲ) we have performed an additional analysis on movement extent as the reviewer suggested. Below, we describe each of these changes in detail. (ⅰ) First, regarding the interpretation of position experience: in the original manuscript, we argued that position experience contributes independently to movement termination, based on the observation that the final position error in the D condition was larger than that in the C condition.

However, as the reviewer correctly pointed out, this interpretation was not directly supported by the data. If position experience independently contributed to termination accuracy, providing position experience in addition to direction experience (i.e., the P&D condition) should improve termination accuracy compared with the D condition. However, we did not observe this benefit at the group level: the final position error in the P&D condition was comparable to that in the D condition (d = −0.14). We therefore no longer claim that position experience contributes independently to movement termination. Instead, we now interpret our results as suggesting that position experience contributes to termination accuracy only when it is integrated with direction experience within a coherent visuomotor representation. The evidence supporting this revised interpretation comes from the alignment analysis, which we describe in our response to Major Comment (3).

Changes in the manuscript (Abstract, p. 1, ll. 16-17): As a result, direction experience improved movement initiation, whereas the lack of position experience was associated with reduced movement termination accuracy. (Significant statement, p. 1, ll. 27-28): direction experience improved movement initiation, while the lack of position experience reduced movement termination accuracy. (Results, p. 15-16, ll. 421-440): These observations were supported by a quantitative comparison of the initial direction error across conditions (Figure 4C). Statistical analysis on the last 5 test trials showed that there was no significant effect of trial, indicating that the learning reached plateau (F(1,370.76)ᵃ = 0.570, p = 0.451), whereas there was a significant main effect of condition (F(3,23.08)ᵇ = 3.98, p = 0.0201). Post hoc pairwise comparisons revealed that the lack of direction experience markedly impaired movement initiation: the initial direction error in the P condition was substantially larger than in the C condition (C − P: t(23)ᶜ = −3.43, p = 0.0136, d = −0.862, 95% CI [−16.9, −1.46]). In contrast, the lack of position experience had little effect on movement initiation. The initial direction error in the D condition was comparable to that in the C condition (C − D: t(23)ᵈ = − 0.861, p = 0.398, d = −0.201, 95% CI [−9.33, 5.04]), and although the P&D condition showed a slightly larger initial direction error than the C condition (C − P&D: t(23)ᵉ = − 1.83, p = 0.162, d = −0.472, 95% CI [−13.0, 2.91]), this difference did not reach statistical significance. Adding position experience to direction experience produced only a small change between the D and P&D conditions (D − P&D: t(23)ᶠ = −1.09, p = 0.346, d = − 0.270, 95% CI [−10.5, 4.77]). Consistent with these patterns, the comparison between the P and D conditions showed a medium effect size in favor of the D condition, although the difference did not reach statistical significance (P − D: t(23)ᵍ = 2.37, p = 0.0790, d = 0.661, 95% CI [−1.52, 15.6]). Altogether, these results suggest that the experience of movement direction contributed to improved accuracy of movement initiation, while the experience of target position had little effect on it. (Results, p. 16-17, ll. 442-473): To evaluate generalization at the termination phase under comparable initial conditions, we focused on the D and P&D conditions, in which the initial direction error was not significantly different from that in the C condition. We further included the initial direction error as a covariate in the statistical model so that the contribution of each condition to movement termination could be evaluated beyond what was already explained by movement initiation. The P condition was excluded from this focused comparison because its initial direction error was substantially larger than that of the C condition, making the termination comparison difficult to interpret under matched initial conditions. Figure 4E shows the distribution of the final cursor position across all four conditions. In the D condition, although the distribution was centered on the target, frequent overshooting occurred. In the P&D condition, the distribution was similarly distributed around the target with frequent deviation. Statistical analysis on the last 5 test trials showed no significant effect of trial, indicating that the learning converged (F(1,365.31)ʰ = 0.172, p = 0.679), whereas there was a significant main effect of condition (F(3,23.28)ⁱ = 7.98, p = 7.81 × 10⁻⁴). The covariate, initial direction error, also showed a strong effect (F(1,436.52)ʲ = 82.97, p < 2.2 × 10⁻¹⁶), confirming that initial direction error was tightly linked to final position error. Post hoc pairwise comparisons revealed that, even when initial direction error was statistically controlled, the final position error in the D condition was substantially larger than in the C condition (C − D: t(23.0)ᵏ = −3.09, p = 0.0106, d = −0.850, 95% CI [−1.86, −0.064]). This indicates that, although the D condition showed comparable movement initiation to the C condition, its movement termination was less accurate. The lack of position experience thus appears to be relevant to the accuracy of movement termination, even when the movement starts from a comparable initial direction. Notably, providing position experience separately, in addition to direction experience, was not sufficient to support accurate movement termination. The final position error in the P&D condition was also significantly larger than in the C condition (C − P&D: t(23.2)ˡ = −4.18, p = 0.0021, d = −0.988, 95% CI [− 1.89, −0.347]), and was comparable to that in the D condition (D − P&D: t(23.0)ᵐ = − 0.529, p = 0.880, d = −0.138, 95% CI [−1.01, 0.696]). These results indicate that, even after experiencing both target position and movement direction in separate training contexts, participants were unable to reach the level of termination accuracy achieved through direct training. What might explain this lack of additive benefit? (Results, p. 17, ll. 476-492): To examine why the additive benefit was not observed at the group level, we focused on the redundancy of the joystick control space. Although position and direction experiences appear additive in the workspace, the same target position can be achieved by infinitely many joystick configurations along the task-irrelevant dimensions (Figure 5A and B). Consequently, the visuomotor outputs derived from direction and position experiences do not necessarily coincide. We therefore quantified the alignment between the direction-based and position-based visuomotor outputs in the joystick control space, defined as the angle between the two outputs. To examine whether this alignment explains individual differences in the final position error in the P&D condition, we performed a linear regression at the participant level with the alignment and initial direction error as predictors of the final position error. After controlling for initial direction error, the alignment was a significant predictor of the final position error (βⁿ = 0.0501, p = 0.00692; Figure 5C), indicating that participants with greater alignment exhibited smaller final position errors in the P&D condition. These results indicate that the alignment between direction- and position-based outputs in the redundant control space underlies how effectively position experience and direction experience are combined to support accurate movement termination. (Discussion, p. 19, ll. 540-542): The results suggested that direction experience enhanced the accuracy of movement initiation, whereas the absence of position experience led to reduced accuracy of movement termination (Figure 4) (Conclusion, p. 23, ll. 653-655): In conclusion, our results show that de novo motor learning relies on both movement direction and target position experiences. Direction experience determined the accuracy of movement initiation, whereas the accuracy of movement termination depended on the alignment between direction- and position-based outputs in the redundant control space. These findings suggest that accurate formation of novel sensorimotor maps requires the coordinated use of directional and positional experiences. (ⅱ) Second, regarding the methodological concern that the final position error is confounded by the initial direction error: we agree with the reviewer that this confound makes it difficult to interpret the contribution of each condition to movement termination, because reaching movements with different initial directions inherently lead to different final positions. To address this, we have included the initial direction error as a covariate in the linear mixed-effects model for the final position error. This allowed us to evaluate the contribution of each condition to movement termination beyond what was already explained by the accuracy of movement initiation. The covariate effect was indeed substantial (F(1, 436.52) = 82.97, p < 2.2 × 10⁻¹⁶), confirming that the initial direction error was tightly linked to the final position error and that controlling for it was necessary. We also focused our comparison on the D and P&D conditions, in which the initial direction error was not significantly different from that in the C condition. The P condition was excluded from this focused analysis because its initial direction error was substantially larger than that of the C condition, which makes the termination comparison difficult to interpret under matched initial conditions. After this adjustment, the final position error in the D condition remained significantly larger than that in the C condition (C − D: t(23.0) = −3.09, p = 0.0106, d = −0.850), indicating that the lack of position experience is associated with reduced accuracy of movement termination even when the movement starts from a comparable initial direction.

Changes in the manuscript: (Methods, p. 10-11, ll. 291-297): ... Finally, to examine whether this alignment explains individual differences in the final position error in the P&D condition while controlling for individual differences in movement initiation, we performed a linear regression at the participant level with the alignment and initial direction error as predictors of the final position error. (Methods, p. 11, ll. 313-318): ... we conducted a linear mixed-effects model analysis using the lme4 package in R (Bates et al., 2015). For the initial direction error, the fixed effects included condition and trial. For the final position error, we additionally included initial direction error as a covariate. This was motivated by the fact that the final cursor position is geometrically influenced by the initial movement direction; statistically controlling for initial direction error therefore allowed us to isolate the contribution of each condition to movement termination beyond what was already explained by movement initiation. (Figure 5C caption, p. 28-29, ll. 800-803): The partial regression plot shows the relationship between output alignment (x-axis) and final position error in the P&D condition (y-axis), adjusted for the initial direction error. Each dot represents an individual participant, the solid line indicates the regression line, and the shaded area denotes its 95% confidence interval. (ⅲ) Third, regarding the reviewer's suggestion that movement extent should be analyzed separately: we performed an additional analysis on the extent error (the absolute deviation between the actual and the ideal movement extent) using the same linear mixed-effects model as for the initial direction error. Contrary to what might have been expected, the analysis revealed no significant main effect of condition on the extent error (F(3, 22.47) = 1.25, p = 0.314). This indicates that, unlike the initial direction error and the final position error, the extent error did not differ systematically across conditions. We therefore did not include this analysis in the main text to keep the focus on the two error measures (initial direction error and final position error). >(2) Repeated-measures experimental design: Another major concern is the experimental design: the authors examine learning here using a generalization paradigm with fully repeated measures where the same participant experiences all 4 conditions in different parts of the workspace. For 'de novo' learning, where participants are learning from scratch, this design seems a bit unconventional because it assumes that participants can learn the 4 directions (which are interleaved during training and have different information) independently and simultaneously. At the very least, this should be followed up by a "between-group" design to show that there is no "interference" between learning one condition vs. learning 4 conditions.

We thank the reviewer for raising this important concern. The within-participant design was a deliberate requirement of our generalization paradigm. The central aim of this paradigm is to evaluate how well learning generalizes to the P, D, and P&D conditions relative to the common baseline provided by the Control (C) condition, in which the test movement is directly trained.

Because de novo learning is characterized by large individual differences, performance has to be evaluated relative to each participant's own C condition. Collecting data from all four conditions in the same participant therefore makes it possible to compare performance within each individual and to isolate the effect of condition from differences in overall learning ability between participants. For this reason, this generalization paradigm essentially requires a within-participant design rather than a comparison across separate groups. To make this rationale explicit to readers, we have also added a description of this central aim to the Methods.

Changes in the manuscript (Methods, p. 8, ll. 203-205):

The aim of this experimental design is to evaluate how well learning generalizes to the P, D, and P&D conditions relative to the common baseline provided by the C condition, in which the test movement is directly trained.

Nevertheless, we cannot rule out the possibility that experience in one condition influenced performance in the other conditions. To directly address the possibility of interference, we conducted an additional experiment (Experiment 2) in which a separate group of 24 participants was trained exclusively on the D (Direction) sequence, matched in sample size to Experiment 1. We focused on the D condition because it provides the central evidence for our main findings: directional experience contributes to the accuracy of movement initiation, whereas the lack of positional experience is associated with reduced accuracy of movement termination. In brief, Experiment 2 reproduced this central dissociation even when the D sequence was trained alone (we report this experiment in a new figure, Figure 6): with sufficient training, the initial direction error in the D condition became comparable to the C condition, whereas the final position error remained larger.

This confirms that the dissociation is not attributable to the interleaving of the four conditions.

Below, we describe the design and results of Experiment 2.

Because Experiment 2 used 20 D sequences per block (vs. 5 per block in Experiment 1), the same amount of practice accumulated within fewer blocks. To align practice experience across conditions and experiments, we defined two matched reference points (Figure 6B): Dmid, the mid-training reference for the D condition (end of block 5; 100 D sequences, matching the total D-sequence experience of the D condition in Experiment 1), and Clast, the last-training reference for the C condition (end of block 10; 100 direct practices of each trained movement, matching the direct practice amount of the C condition in Experiment 1). In addition, to examine the effect of further directional training, we defined a late-training reference for the D condition, Dlast (end of block 10;

200 D sequences). All comparisons used the five test trials at the corresponding stage of training.

At the matched-practice point, the initial direction error in Dmid was significantly larger than in Clast (Clast − Dmid: t(23) = −4.05, p = 0.0005, d = −0.99, 95% CI [−16.0, −5.16]; Figure 6C). Thus, at this matched-practice point Experiment 2 did not reproduce the comparability between the D and C conditions observed in Experiment 1. However, with continued training, the initial direction error in Dlast converged to a level comparable to Clast (Clast − Dlast: t(23) = −1.49, p = 0.150, d = −0.21, 95% CI [−4.71, 0.77]; Figure 6D), with an effect size that closely matches the C − D comparison in Experiment 1 (d = −0.201). A direct between-experiment comparison showed no significant difference, although the error tended to be smaller in Experiment 1 (D (Exp1) − Dmid (Exp2): t(46) = −1.80, p = 0.079, d = −0.60, 95% CI [−13.6, 0.77]; Figure 6E).

The final position error in Dmid was larger than in Clast (Clast − Dmid: t(25.9) = −6.27, p < 0.0001, d = −1.63, 95% CI [−2.70, −1.37]; Figure 6F), reproducing the impaired termination under directional experience alone. This error remained larger than Clast even after continued training (Clast − Dlast: t(23.1) = −5.09, p < 0.0001, d = −2.09, 95% CI [−3.02, −1.27]; Figure 6G), indicating that additional practice did not improve termination. The between-experiment comparison again showed no significant difference, although the error tended to be smaller in Experiment 1 (D (Exp1) − Dmid (Exp2): t(46.6) = −0.901, p = 0.372, d = −0.301, 95% CI [−1.4, 0.532]; Figure 6H).

Taken together, these results confirm that the central dissociation of Experiment 1 is reproduced and is not attributable to interleaving the four conditions: with sufficient D-sequence training, directional experience alone was sufficient to achieve accurate movement initiation (the initial direction error in Dlast reached a level comparable to Clast; Figure 6D), but not accurate movement termination (the final position error in Dlast remained larger than Clast; Figure 6G). At the same time, we acknowledge that interleaving produced interference: in Experiment 1, both the initial direction error and the final position error tended to decrease faster (Figure 6E and H), possibly because participants spent more total time engaged in the task across the interleaved conditions. Importantly, however, this influence was limited to the rate of learning and did not change what could ultimately be learned, and therefore does not affect the central dissociation described above.

Changes in the manuscript:

We added a new "Experiment 2" section to the Methods (Participants, Task, Data analysis, and Statistics) and a new Results subsection ("Generalization in the D condition under single-condition training"), together with the new Figure 6. (Methods, p. 12-14, ll. 332-391; Results, p. 17-18, ll.

494-534; Figure 6 Caption, p. 29, ll. 805-823) Figure 6. A: Training targets consisted of five targets, T1-T5 (left); the test target was located in the direction matching the trained movements (T2→T3 and T4→T5) (right). The assignment of the D condition to one of the four cardinal test directions was counterbalanced across participants; the configuration shown is one example. B: The task consisted of 10 blocks; each block contained 20 D sequences (magenta) and 8 test trials (gray) randomly interleaved. C: Initial direction error, Clast vs.

Dmid. D: Initial direction error, Clast vs. Dlast. E: Initial direction error in the D condition, Experiment 1 vs. Experiment 2 (Dmid). F: Final position error, Clast vs. Dmid. G: Final position error, Clast vs. Dlast.

H: Final position error in the D condition, Experiment 1 vs. Experiment 2 (Dmid). In C-H, individual dots connected by lines represent each participant's mean error.

Relatedly, this experimental design assumes that all parts of the workspace are "equal" - however the PC analysis typically has an "easier' PC1 relative to PC2 (depending on the relative variances accounted by the PCs) - in other words, control along the X and Y axes may not be 'equal' in this task and there may also be biomechanical factors that drive the relative ease of the different directions (Fig 3 hints at some of this, where the movement time is not the same across all 4 conditions even initially in learning; but no statistics are provided here). The PC data could be helpful in assessing it. Alternatively, a control experiment where the positions of the P, C, D and P&D altered could be used to show that these effects are not artifacts of biomechanical constraints or the PCs Regarding the concern about the condition assignment to the workspace: Thank you for raising this important point. We would like to clarify that the assignment of experimental conditions to the four target directions was counterbalanced across participants, as described in the Methods section. To make this clearer, we have added "An example configuration" to the Figure 2C and updated the caption to explicitly note that the shown assignment is one example of the counterbalanced arrangements.

Changes in the manuscript (Figure 2 Caption, p. 27, ll. 756-757):

The assignment of conditions to the four test targets (C, P, D, P&D) was counterbalanced across participants; the configuration shown here is one example. >(3) Redundancy analyses - the authors clearly use a redundant task here - yet, no analysis of the redundancy is performed (especially to check if the use of the redundant space changed with condition). This could provide more insight into the controller rather than simply looking at task performance, and could bolster their final conclusion of the correlations in the P&D condition.

We sincerely thank the reviewer for this insightful comment. We would first like to note that the alignment analysis is itself an analysis of how participants used the redundancy of the control space, as it characterizes how the direction- and position-based visuomotor outputs are organized within the task-irrelevant dimensions of the joystick control space. We agree that the redundancy of the control space is a key feature of this de novo learning task and deserves more explicit treatment. In the revised manuscript, we have reframed the alignment analysis as an analysis of how participants used the redundant control space, and we have positioned this analysis as a central piece of evidence supporting our revised interpretation of position experience.

Specifically, the six-dimensional joystick state is mapped onto the two-dimensional cursor position, leaving four task-irrelevant dimensions. Within this redundant control space, the joystick states inferred from direction and position experience do not necessarily coincide. By quantifying the relationship between these two visuomotor outputs, the alignment analysis directly characterizes how participants used the redundancy. This perspective is central to our revised interpretation: as described in our response to Major comment 1, position experience contributes to movement termination not independently, but only when integrated with direction experience within a coherent visuomotor representation. The alignment analysis provides direct empirical support for this view, since participants with greater alignment exhibited smaller final position errors in the P&D condition (Figure 5C). To make this framing explicit, we revised the heading and opening of the corresponding Results subsection (Use of the redundant control space relates to generalization at movement termination), as shown below.

Changes in the manuscript (Results, p. 17, ll. 476-477):

To examine why the additive benefit was not observed at the group level, we focused on the redundancy of the joystick control space.

Other comments >At several points, the authors indicate that there was "No effect of trial, indicating learning has plateaued". This statement is not clear given the clear learning curve presented in Fig 4 that shows that errors decrease with trials. Please clarify in the Results that only the last 5 trials were used for analysis.

We thank the reviewer for pointing out this lack of clarity. We have now explicitly clarified in the Results section that the statistical analyses showing no effect of trial were performed specifically on the last 5 test trials.

Changes in the manuscript: (Results, p. 15, ll. 421): These findings were supported by a quantitative comparison of the initial direction error across conditions (Figure 4C). Statistical analysis on the last 5 test trials showed that there was no significant effect of trial, indicating that the learning reached plateau. (Results, p. 16, ll. 453): We compared the final position error across conditions. Statistical analysis on the last 5 test trials showed that there was no significant effect of trial, indicating that the learning converged. >Please indicate if the joysticks are spring-loaded (i.e., they return to the center when the fingers are removed) We thank the reviewer for pointing this out. We have now explicitly indicated in the "Experimental setup" section that the joysticks are spring-loaded and return to the center when the fingers are removed.

Changes in the manuscript (Methods, p. 4, ll. 109-111):

Each joystick measured 3-axis positions (i.e., X: horizontal, Y: vertical, and Z: roll axis), acquiring a total of 6 input signals in 16-bit at a sampling rate of 50 Hz. Both joysticks were spring-loaded, meaning they passively returned to the center position when the fingers were removed. We performed preprocessing on these inputs in real time... >Please provide some indication of what the PC loadings looked like across participants, especially if the mappings generally resulted in "independent' hand controls (i.e. one joystick controls X, the other controls Y etc.) or if they were typically coupled.

Thank you for this insightful suggestion. We have added Figure 1-1 (Extended Data) showing the mapping matrices for all participants. The PC loadings typically involved coordinated contributions from both joysticks rather than independent hand control, though the specific patterns varied across participants. We have added a reference to this Extended Data figure in the Calibration task section of the Methods.

Changes in the manuscript (Methods, p. 5, ll. 137): ... in the following reaching task. Individual mapping matrices for all participants are shown in Figure 1-1. >Figure 4 show a large number of data points on the box plots/scatter plots (even though there are only 24 participants). Within-participant data should not be displayed at the same level as between participant data since they represent different things and can be misleading).

We thank the reviewer for this important point. We completely agree that pooling all trials obscures the distinction between within-participant and between-participant variability. To address this issue, we have revised both the scatter plots (Figure 4B, 4E) and the boxplots (Figure 4C, 4F) to display only the participant-level data. Specifically, the data points in the revised scatter plots now represent the mean cursor position of the last 5 test trials for each of the 24 participants, and the confidence ellipses are fitted to these participant means. Similarly, the revised boxplots now show the mean error for each participant, connected by lines to explicitly illustrate the repeated-measures design.

We have updated the figure captions accordingly.

Changes in the manuscript (Caption in Figure 4, p. 28, ll. 777-787):

Figure 4. Initial direction error and final position error. A: Initial direction error across test trials. Thick lines indicate the mean across participants. Error bars indicate mean {plus minus} standard error across participants. B: Distribution of cursor positions at movement initiation. The dots represent the mean cursor position of the last 5 test trials for each of the 24 participants. Ellipses indicate 68% confidence intervals. C: The boxplot of initial direction error. The individual dots connected by lines represent the mean error of the last 5 test trials for each of the 24 participants. D: Final position error across test trials. Thick lines indicate the mean across participants. Error bars indicate mean {plus minus} standard error across participants. E: Distribution of cursor positions at movement termination. The dots represent the mean cursor position of the last 5 test trials for each of the 24 participants.

Ellipses indicate 68% confidence intervals. F: The boxplot of final position error. The individual dots connected by lines represent the mean error of the last 5 test trials for each of the 24 participants. >The inference for "Alignment" may be better captured by the angle between VOdir and VOpos (rather than the magnitude of the difference vector) Thank you for this suggestion. Following your recommendation, we have recalculated the alignment using the angle between VOdir and Vopos instead of the norm of the difference vector. The significant correlation was preserved, supporting our original interpretation. We have updated Figure 5C accordingly.

Changes in the manuscript (Methods, p. 10-11, ll. 291-297): ... estimates of these averages. To quantify the alignment between the two outputs, we calculated the angle between VOdir and VOpos as arccos((VOdir · VOpos) / (ǁVOdirǁ × ǁVOposǁ)), where a smaller value indicates greater alignment. >The discussion about "Neural representations" seems far too speculative to tie directly to the results here. At the very least, it can be shortened and focused on how this actually relates to the conclusions of this study We thank the reviewer for this important point. We agree that the previous version of this section may have overextended the interpretation of our behavioral findings to specific neural substrates. In response, we have substantially shortened this section and refocused it on the conceptual link between our findings and established distinctions in spatial representation, rather than on detailed neurophysiological claims. We now frame the egocentric-allocentric distinction as a plausible interpretative framework that is consistent with our results, rather than as a direct account of the underlying neural mechanisms.

Change in manuscript (Discussion, p. 22-23, ll. 640-650):

Direction- and position-based learning may rely on distinct representations of the target, such as a cursor-centered displacement vector specifying movement direction and an absolute target position anchored in external space. These two forms of information are broadly consistent with the distinction between allocentric and egocentric spatial representations (Chen and Crawford, 2020; Milner and Goodale, 2008; Schenk, 2006).

From this perspective, the two forms of learning observed in our study can be interpreted in relation to these spatial codes: position-based learning may reflect reliance on egocentric representations of target location, whereas direction-based learning may reflect reliance on allocentric representations of the spatial relation between the cursor and the target. This interpretation is consistent with the idea that multiple spatial representations can be maintained in parallel to support flexible visuomotor behavior.

Additional corrections during revision During the revision process, we identified several points in the manuscript that required correction.

These corrections, which were not explicitly pointed out by the reviewers, are described below, and the corresponding changes in the manuscript are highlighted in green.

While revising the manuscript, we identified a minor inconsistency in the analysis code for computing the initial direction error. In the original analysis, the peak of the joystick tangential velocity was searched across the entire trial duration, which occasionally detected small velocity peaks occurring before movement onset. We have corrected this by restricting the peak detection to the interval after movement onset, consistent with the intended procedure described in the Methods.

We re-ran all analyses involving the initial direction error with this corrected procedure. The overall pattern of results and main conclusions were preserved, though specific statistical values were slightly updated. The Methods section has been revised to clarify this point and all affected results (Figure 4A-C, Table 1) and statistical values have been updated accordingly.

Change in manuscript (Methods, p. 10, ll. 270):

The initial direction error was quantified as the absolute angle between the direction from the center of the workspace to the cursor position at the time when the joystick tangential velocity reached its first peak after movement onset and the direction from the center of the workspace to the target position.

## References

[B1] Albert ST, Hadjiosif AM, Jang J, Zimnik AJ, Soteropoulos DS, Baker SN, Churchland MM, Krakauer JW, Shadmehr R (2020) Postural control of arm and fingers through integration of movement commands. Elife 9:e52507. 10.7554/eLife.52507 PMC7062460 32043973

[B2] Bates D, Mächler M, Bolker B, Walker S (2015) Fitting linear mixed-effects models using lme4. J Stat Softw 67:1–48. 10.18637/jss.v067.i01

[B3] Berger DJ, Gentner R, Edmunds T, Pai DK, d’Avella A (2013) Differences in adaptation rates after virtual surgeries provide direct evidence for modularity. J Neurosci 33:12384–12394. 10.1523/JNEUROSCI.0122-13.2013 PMC6618678 23884944

[B4] Berger DJ, Borzelli D, d’Avella A (2022) Task space exploration improves adaptation after incompatible virtual surgeries. J Neurophysiol 127:1127–1146. 10.1152/jn.00356.2021 35320031

[B5] Chen Y, Crawford JD (2020) Allocentric representations for target memory and reaching in human cortex. Ann N Y Acad Sci 1464:142–155. 10.1111/nyas.14261 31621922

[B6] Choi Y, Shin EY, Kim S (2020) Spatiotemporal dissociation of fMRI activity in the caudate nucleus underlies human de novo motor skill learning. Proc Natl Acad Sci U S A 117:23886–23897. 10.1073/pnas.2003963117 PMC7519330 32900934

[B7] Dal’Bello LR, Izawa J (2021) Task-relevant and task-irrelevant variability causally shape error-based motor learning. Neural Netw 142:583–596. 10.1016/j.neunet.2021.07.015 34352492

[B8] Donchin O, Francis JT, Shadmehr R (2003) Quantifying generalization from trial-by-trial behavior of adaptive systems that learn with basis functions: Theory and experiments in human motor control. J Neurosci 23:9032–9045. 10.1523/JNEUROSCI.23-27-09032.2003 PMC6740843 14534237

[B9] Georgopoulos AP, Kalaska JF, Massey JT (1981) Spatial trajectories and reaction times of aimed movements: Effects of practice, uncertainty, and change in target location. J Neurophysiol 46:725–743. 10.1152/jn.1981.46.4.725 7288461

[B10] Ghez C, Scheidt R, Heijink H (2007) Different learned coordinate frames for planning trajectories and final positions in reaching. J Neurophysiol 98:3614–3626. 10.1152/jn.00652.2007 17804576

[B11] Gibson JJ (1979) *The ecological approach to visual perception*. Boston: Houghton Mifflin.

[B12] Haith AM, Yang CS, Pakpoor J, Kita K (2022) De novo motor learning of a bimanual control task over multiple days of practice. J Neurophysiol 128:982–993. 10.1152/jn.00474.2021 36129208

[B13] Jacobs DM, Michaels CF (2007) Direct learning. Ecol Psychol 19:321–349. 10.1080/10407410701432337

[B14] Kawano T, Kouzaki M, Hagio S (2024) Generalization in de novo learning of virtual upper limb movements is influenced by motor exploration. Front Sports Act Living 6:1370621. 10.3389/fspor.2024.1370621 PMC10950898 38510523

[B15] Kim J, Park S, Yoo K, Kim S (2024) Double dissociation of visuomotor interaction mediated by visual feedback during continuous de novo motor learning. Commun Biol 7:1117. 10.1038/s42003-024-06808-z PMC11391080 39261584

[B16] Krakauer JW, Pine ZM, Ghilardi MF, Ghez C (2000) Learning of visuomotor transformations for vectorial planning of reaching trajectories. J Neurosci 20:8916–8924. 10.1523/JNEUROSCI.20-23-08916.2000 PMC6773094 11102502

[B17] Krakauer JW, Hadjiosif AM, Xu J, Wong AL, Haith AM (2019) Motor learning. Compr Physiol 9:613–663. 10.1002/j.2040-4603.2019.tb00069.x 30873583

[B18] Kurtzer I, Herter TM, Scott SH (2005) Random change in cortical load representation suggests distinct control of posture and movement. Nat Neurosci 8:498–504. 10.1038/nn1420 15768037

[B19] Liu X, Scheidt RA (2008) Contributions of online visual feedback to the learning and generalization of novel finger coordination patterns. J Neurophysiol 99:2546–2557. 10.1152/jn.01044.2007 18353914

[B20] Malfait N, Shiller DM, Ostry DJ (2002) Transfer of motor learning across arm configurations. J Neurosci 22:9656–9660. 10.1523/JNEUROSCI.22-22-09656.2002 PMC6757833 12427820

[B21] Milner AD, Goodale MA (2008) Two visual systems re-viewed. Neuropsychologia 46:774–785. 10.1016/j.neuropsychologia.2007.10.005 18037456

[B22] Mosier KM, Scheidt RA, Acosta S, Mussa-Ivaldi FA (2005) Remapping hand movements in a novel geometrical environment. J Neurophysiol 94:4362–4372. 10.1152/jn.00380.2005 16148276

[B23] Park S, Kim J, Kim S (2024) Corticostriatal activity related to performance during continuous de novo motor learning. Sci Rep 14:3731. 10.1038/s41598-024-54176-9 PMC10867026 38355810

[B24] Ranganathan R, Adewuyi A, Mussa-Ivaldi FA (2013) Learning to be lazy: Exploiting redundancy in a novel task to minimize movement-related effort. J Neurosci 33:2754–2760. 10.1523/JNEUROSCI.1553-12.2013 PMC3711630 23407935

[B25] Sadtler PT, Quick KM, Golub MD, Chase SM, Ryu SI, Tyler-Kabara EC, Yu BM, Batista AP (2014) Neural constraints on learning. Nature 512:423–426. 10.1038/nature13665 PMC4393644 25164754

[B26] Scheidt RA, Ghez C (2007) Separate adaptive mechanisms for controlling trajectory and final position in reaching. J Neurophysiol 98:3600–3613. 10.1152/jn.00121.2007 17913996

[B27] Schenk T (2006) An allocentric rather than perceptual deficit in patient D.F. Nat Neurosci 9:1369–1370. 10.1038/nn1784 17028584

[B28] Shadmehr R (2004) Generalization as a behavioral window to the neural mechanisms of learning internal models. Hum Mov Sci 23:543–568. 10.1016/j.humov.2004.04.003 PMC2722915 15589621

[B29] Shadmehr R (2017) Distinct neural circuits for control of movement vs. holding still. J Neurophysiol 117:1431–1460. 10.1152/jn.00840.2016 PMC5376603 28053244

[B30] Shadmehr R, Mussa-Ivaldi FA (1994) Adaptive representation of dynamics during learning of a motor task. J Neurosci 14:3208–3224. 10.1523/JNEUROSCI.14-05-03208.1994 PMC6577492 8182467

[B31] Soechting JF, Lacquaniti F (1983) Modification of trajectory of a pointing movement in response to a change in target location. J Neurophysiol 49:548–564. 10.1152/jn.1983.49.2.548 6834087

[B32] Van der Graaff MCW, Brenner E, Smeets JBJ (2017) Vector and position coding in goal-directed movements. Exp Brain Res 235:681–689. 10.1007/s00221-016-4828-9 PMC5315739 27858127

[B33] Van Sonderen JF, Denier van der Gon JJ, Gielen CC (1988) Conditions determining early modification of motor programmes in response to changes in target location. Exp Brain Res 71:320–328. 10.1007/BF00247492 3169167

[B34] Wang J, Sainburg RL (2005) Adaptation to visuomotor rotations remaps movement vectors, not final positions. J Neurosci 25:4024–4030. 10.1523/JNEUROSCI.5000-04.2005 PMC6724955 15843604

[B35] Wu HG, Smith MA (2013) The generalization of visuomotor learning to untrained movements and movement sequences based on movement vector and goal location remapping. J Neurosci 33:10772–10789. 10.1523/JNEUROSCI.3761-12.2013 PMC4469867 23804099

[B36] Yang CS, Cowan NJ, Haith AM (2021) De novo learning versus adaptation of continuous control in a manual tracking task. Elife 10:e62578. 10.7554/eLife.62578 PMC8266385 34169838

